# Publisher Correction: Targeting Toll-like receptor-driven systemic inflammation by engineering an innate structural fold into drugs

**DOI:** 10.1038/s41467-023-42294-3

**Published:** 2023-10-13

**Authors:** Ganna Petruk, Manoj Puthia, Firdaus Samsudin, Jitka Petrlova, Franziska Olm, Margareta Mittendorfer, Snejana Hyllén, Dag Edström, Ann-Charlotte Strömdahl, Carl Diehl, Simon Ekström, Björn Walse, Sven Kjellström, Peter J. Bond, Sandra Lindstedt, Artur Schmidtchen

**Affiliations:** 1 Division of Dermatology and Venereology, Department of Clinical Sciences, Lund University, SE-22184 Lund, Sweden; 2 Bioinformatics Institute (BII), Agency for Science, Technology and Research (A*STAR), Singapore, 138671 Singapore; 3 Department of Clinical Sciences, Lund University, SE-22184 Lund, Sweden; 4 Department of Cardiothoracic Surgery, Anesthesia and Intensive Care, Skåne University Hospital, SE-22185 Lund, Sweden; 5 SARomics Biostructures AB, Medicon Village, SE-22381 Lund, Sweden; 6 BioMS - Swedish National Infrastructure for Biological Mass Spectrometry, SE-22184 Lund, Sweden; 7 Division of Mass Spectrometry, Department of Clinical Sciences, Lund University, SE-22184 Lund, Sweden; 8 Department of Biological Sciences, National University of Singapore, Singapore, 117543 Singapore; 9 Dermatology, Skane University Hospital, SE-22185 Lund, Sweden

**Keywords:** Sepsis, Drug discovery and development

Correction to: *Nature Communications* 10.1038/s41467-023-41702-y, published online 29 September 2023

The original version of this Article contained an error in Fig. 4 and the corresponding legend in which panel b incorrectly omitted a complete description of *p*-values, and data points were missing to reflect the raw data provided in the Source Data file. This has been corrected in both the PDF and HTML versions of the Article.

