# Supplementary material for: Targeting Toll-like receptor-driven systemic inflammation by engineering an innate structural fold into drugs
Source: Nat Commun. 2023 Sep 29;14:6097. doi: 10.1038/s41467-023-41702-y (PMC10541425; doi:10.1038/s41467-023-41702-y)
Supplement: Supplementary file 1 — Supplementary Information [file 41467_2023_41702_MOESM1_ESM.pdf]

Targeting Toll-like receptor-driven systemic inflammation by engineering an innate structural fold into drugs

Ganna Petruk[1,10,*], Manoj Puthia[1,10], Firdaus Samsudin[2], Jitka Petrlova[1], Franziska Olm[3], Margareta Mittendorfer[3], Snejana Hyllén[3,4], Dag Edström[3,4], Ann-Charlotte Strömdahl[1], Carl Diehl[5], Simon Ekström[6], Björn Walse[5], Sven Kjellström[7], Peter J. Bond[2,8], Sandra Lindstedt[3,4] and Artur Schmidtchen[1,9]

[1]Division of Dermatology and Venereology, Department of Clinical Sciences, Lund University, SE-22184 Lund, Sweden.

[2]Bioinformatics Institute (BII), Agency for Science, Technology and Research (A*STAR), Singapore 138671, Singapore.

[3]Department of Clinical Sciences, Lund University, SE-22184 Lund, Sweden.

[4]Department of Cardiothoracic Surgery, Anesthesia and Intensive Care, Skåne University Hospital, SE-22185 Lund, Sweden.

[5]SARomics Biostructures AB, Medicon Village, SE-22381 Lund, Sweden.

[6]BioMS - Swedish National Infrastructure for Biological Mass Spectrometry, SE-22184 Lund, Sweden.

[7]Division of Mass Spectrometry, Department of Clinical Sciences, Lund University, SE-22184 Lund, Sweden.

[8]Department of Biological Sciences, National University of Singapore, Singapore 117543, Singapore.

[9]Dermatology, Skane University Hospital, SE-22185 Lund, Sweden.

[10]These authors contributed equally: Ganna Petruk, Manoj Puthia

[*]To whom correspondence should be addressed: Ganna Petruk, ganna.petruk@med.lu.se

Keywords: stapling, drug design, host defense peptide, anti-inflammatory, thrombin, sepsis

Supplementary Information

Supplementary Notes:

Supplementary Note 1: Selection of stapling position pag. 1

Supplementary Note 2: NMR analysis of sHVF18 structure pag. 2

Supplementary Note 3: Molecular simulations of sHVF18-CD14 interaction pag. 4

Supplementary Note 4: Molecular simulations of the sHVF18-lipid A and sHVF18-LPS interaction pag. 4

Supplementary Tables:

Supplementary Table 1 pag. 6

Supplementary Table 2 pag. 7

Supplementary Table 3 pag. 8

Supplementary Table 4 pag. 9

Supplementary Table 5 pag. 10

Supplementary Table 6 pag. 11

Supplementary Table 7 pag. 12

Supplementary Table 8 pag. 13

Supplementary Figures:

Supplementary Figure 1 pag. 14

Supplementary Figure 2 pag. 15

Supplementary Figure 3 pag. 17

Supplementary Figure 4 pag. 18

Supplementary Figure 5 pag. 18

Supplementary Figure 6 pag. 19

Supplementary Figure 7 pag. 20

Supplementary Figure 8 pag. 21

Supplementary Figure 9 pag. 21

Supplementary Figure 10 pag. 22

Supplementary Figure 11 pag. 23

Supplementary Figure 12 pag. 24

Supplementary Figure 13 pag. 25

Supplementary Figure 14 pag. 26

Supplementary Figure 15 pag. 27

Supplementary Figure 16 pag. 28
Supplementary Figure 17 pag. 29
Supplementary Figure 18 pag. 30
Supplementary Figure 19 pag. 32
Supplementary Figure 20 pag. 33
Supplementary Figure 21 pag. 34
Supplementary Figure 22 pag. 35
Supplementary Figure 23 pag. 36
Supplementary Figure 24 pag. 36

Supplementary References:

Supplementary References pag. 37

Supplementary Notes

Supplementary Note 1: Selection of stapling position

The position of stapling of GKY25 was based on a design strategy using structural, functional, and evolutionary data (Fig. 1a) in combination with *in silico* analysis of the predicted CD14 binding energies. The centrally located lysine residues (K13, K14, and K18) are important for GKY25's antimicrobial activity[1, 2]. Furthermore, protonation of H8 at pH 5.5 increases the antibacterial activity of GKY25 against Gram-negative *E. coli* by membrane disruption[3]. GKY25 binds to LPS, and the LPS-binding hydrophobic pocket of CD14 and the residues responsible for LPS and CD14 interaction have been defined[2]. Studies demonstrate that K14 cross-links to K87 in CD14, and *in silico* docking studies show that the C-terminal residues of Q17, K18, D21, Q22, and E25 are exposed to the solvent[2]. NMR studies have determined the LPS-bound conformation (PDB:5Z5X [https://www.rcsb.org/structure/5Z5X]) in which the C-terminal α-helix starts at I16. Interactions with LPS are mediated with hydrophobic residues and the positively charged residues H8, R11, K13, and K14[2]. Based on these data, the only suitable amino acid residues remaining available for stapling were G1, L12, Q17, D21, Q22, and E25.

We then performed an *in silico* analysis of peptide staple positions, whereby a short hydrophobic pentenyl-alanine staple linking either residue i and i+3 or residues i and i+4 was added along the sequence of GKY25. To determine the effect of adding this staple upon binding to CD14, we then calculated the difference in binding energies between the non-stapled and stapled version of the peptide (Supplementary Fig. 1a) (see details in Methods). We found that the addition of a staple at most positions, particularly on the N-terminal region of the peptide, resulted in poorer binding, as demonstrated by the positive binding energy differences. The reduced affinities could be caused by the staple perturbing interactions between the peptide and CD14. A few staples resulted in improved binding to CD14. For the i-i+3 configuration, these include I16-V19, V19-Q22, I20-F23, and D21-G24, whereas for the i-i+4 configuration, these include V9-K13 and Q17-D21. All the staples that resulted in a more favorable binding to CD14 comprise hydrophobic residues, which could be important for interaction with LPS, except the Q17-D21 staple. Taken together, we, therefore, opted for stapling in the position of Q17 and D21.

We first analyzed the helicity of stapled GKY25 (herein denoted as sGKY25) in comparison to its linear version by using circular dichroism (CD) (Supplementary Fig. 2a). The spectrum of GKY25 in the presence of LPS was used as a positive control. CD analysis of sGKY25 was compatible with an α-helical structure, and the content of the helicity was

comparable to GKY25 when it is bound to LPS. In the case of sGKY25, the α-helical content remained unchanged upon LPS binding. To assess whether stapling increased the proteolytic stability of the peptide, we exposed it to various proteases for different lengths of time, followed by analysis by SDS-PAGE. As shown in Supplementary Fig. 2b, stapling enhanced protease resistance in the presence of HNE for up to 6 h. Protease resistance to trypsin was also increased, with detectable intact sGKY25 after 6 h of digestion. Interestingly, neither of the two peptides showed susceptibility to V8, an enzyme specifically targeting peptide bonds on the carboxyl-terminal side of either aspartate or glutamate [4].

We next employed LC-MS/MS to understand which regions were released from the digested sGKY25 in comparison to the linear peptide. The results are summarized in Supplementary Fig. 2c and Supplementary Table 1. As expected, the linear non-stapled peptide was extensively fragmented already after 30 min of digestion. Stapling yielded partial protection of sGKY25, particularly in the C-terminal part of the peptide. Notably, stapling of GKY25 increased the cytotoxicity of GKY25 against human monocytes (Supplementary Fig. 2d). Using LPS-stimulated human blood, sGKY25 showed significantly less reduction of TNF-α and IL-ß when compared with the original linear peptide (Supplementary Fig. 2e). sGKY25 was significantly more hemolytic towards red blood cells (RBCs) (Supplementary Fig. 2f). These findings, together with the observation that sGKY25 was only partially protease-resistant, precluded any further development of sGKY25, motivating evaluation of additional variants containing the structurally constrained CD14 interacting region.

Inspection of the *in silico* data showed that structural locking of G1-F5 was compatible with retained CD14 interaction (Supplementary Fig. 1), and as this would also confer possible protection from aminopeptidases, we, therefore, designed a double stapled GKY25 (denoted 2sGKY25). For this, F5 was replaced by E5, enabling a lactam bridge formation. Moreover, we observed that shorter peptides, corresponding to the original sequence HVFRLKKWIQKVIDQFGE (HVF18) [1], were formed during the proteolysis of sGKY25. HVF18 is generated by neutrophil elastase in wounds [5], and its mode of action includes interactions with LPS as well as the LPS-binding groove of CD14 via its critical KKWIQK region [2]. With these background data, we, therefore, decided to analyze stapled HVF18 and related N-terminally truncated stapled versions containing the CD14-interacting region.

Supplementary Note 2: NMR analysis of sHVF18 structure

Initial NMR experiments were performed with sHVF18 dissolved in DMSO-d6. We obtained several NOE cross-peaks that allowed us to assign around 60% of the whole sequence non-ambiguously (Supplementary Fig. 5a-d and Supplementary Table 3). Notably, the Hα-Cα

peaks in ^{13}C-HSQC and HN-N amide backbone peaks in the ^{15}N-SOFAST-HMQC are missing likely due to the oligomerization of the peptide. We, therefore, dissolved the peptide in TFE, which is known to stabilize the secondary structure of peptides [6]. ^{1}H and TOCSY spectra for sHVF18 in 25% and 50% TFE were overlaid (Supplementary Fig. 6a-b) with similar peak positions and peak shapes for both samples. The 50% TFE sample was selected for further measurements based on the peak width for the stapled peptide linker (Supplementary Fig. 7a-e, respectively). Data were collected for the sHVF18 in 50% TFE. TOCSY, NOESY, and ROESY spectra (Supplementary Fig. 7a-c, respectively) showed well-dispersed peaks, where amino acid type can be easily identified in the TOCSY spectra. NOESY and ROESY spectra show many HN-Hα and HN-HN cross peaks, allowing an easy sequential assignment of the peptide. The ^{13}C HSQC spectrum (Supplementary Fig. 7d) shows well-dispersed peaks. Sixteen cross peaks corresponding to amide backbone atoms could be detected in the ^{15}N SOFAST-HMQC spectra (Supplementary Fig. 7e), as well as side-chain cross peaks for W8 and Q15. The ^{15}N HMQC and ^{13}C HSQC spectra indicate that sHVF18 presents a well-defined conformational state under these conditions. The presence of multiple HN-HN (I,i+2) and HN-Hα (I, i+2/3) signals indicate a well-defined secondary structure. Assignments were performed, and 97% of the available ^{1}H resonances could be identified (Supplementary Tables 3 and 4). The DANGLE dihedral angle estimations and chemical shift index (CSI) module in the CCPNMR suite estimated that sHVF18 contains an α-helix consisting of residues 7 to 14, which is also consistent with the NOE pattern. The stapled linker is easily seen in the NOESY spectra due to the aromaticity of the staple (Supplementary Fig. 8) and based on TOCSY spectra, the presence of the staple can be confirmed. Due to the significant peak overlap and complexity of the TOCSY spectra, inter-residue NOE cross peaks of neighbor residues were used to identify the sequence of the stapled residues. The sHVF18 3D structure ensemble converged to a backbone RMSD of 0.61 Å. Statistics for sHVF18 are presented in Supplementary Table 5. RMSD for the backbone and for all atoms are shown in Supplementary Table 6.

A comparison of NOESY spectra for sHVF18 in 50% TFE and DMSO-d6, after the assignment of sHVF18 in 50% TFE had been done, indicates that sHVF18 in DMSO-d6 is at least partially folded. This can especially be seen for the HN-HN correlations in the amide region of the NOESY spectra which is indicative of α-helical secondary structures. In addition, cross-peaks are detected between the linker and the amide and aromatic region of the NOESY spectra, further indicating the presence of a folded structure. However, line broadening is severe for sHVF18 in the DMSO-d6 spectra, which is indicative of conformational exchange and prohibiting full assignment.

Supplementary Note 3: Molecular simulations of sHVF18-CD14 interaction. We performed all-atom MD simulations of sHVF18 bound to CD14 to understand the effect of peptide binding on the dynamics of CD14. Interestingly, our HDX-MS experiments identified regions distal from the N-terminus of CD14 that exhibit deuterium exchange protection upon sHVF18 binding, for example, residues L123-W160. To investigate any potential long-range allosteric effects of peptide binding on the conformation of CD14, we performed simulations of *apo* CD14 and compared the per-residue root mean square fluctuations (RMSFs) as a measure of flexibility (Supplementary Fig. 14e and f). As expected, a significant decrease in RMSF was observed in residues proximal to the peptide binding site on the N-terminus of CD14 (residues 42-52 and 71-82), consistent with deuterium exchange protection observed in HDX. However, most residues outside of the binding site show overlapping RMSF values, indicating no changes in dynamics during the timescale of our simulations. Longer simulations are likely required to sample any allosteric conformational changes due to peptide binding on CD14.

Supplementary Note 4: Molecular simulations of the sHVF18-lipid A interaction.
Our HDX-MS data demonstrate weakened protection of the putative LPS binding site on the N-terminus of CD14 from deuterium exchange in the presence of sHVF18 and LPS, compared to experiments with LPS alone (Fig. 3c). While our *in silico* modeling and simulations suggest a competitive binding of the peptide and LPS at the N-terminal rim of CD14 (Fig. 3d and e), another possible mechanism of action is direct neutralization of LPS in solution by sHVF18. Previously, we showed that other similar TCPs, such as GKY25, HVF18, and VFR12, can adsorb and disperse on the surface of lipid A micelles [2]. Lipid A represents the primary bioactive lipid component of LPS; thus, these peptides could encase LPS aggregates in solution and prevent them from interacting with CD14. To investigate if this characteristic is preserved in the stapled peptide, we performed microsecond timescale coarse-grained (CG) MD simulations of sHVF18 with a lipid A aggregate at a 1:2 ratio of peptide to lipid. The simulations included Ca^{2+} since divalent cations are crucial to cross-link neighboring phosphate headgroups of lipid A. We found that in all three independent 10 μs simulations, sHVF18 adsorbed onto the surface of the lipid A aggregate and covered a significant portion of the surface, predominantly the exposed lipid tails (Supplementary Fig. 15a). To quantify the degree of surface burial by sHVF18, we measured SASA of the lipid headgroups and lipid tails throughout the simulations and compared it to simulations of a lipid A aggregate without sHVF18 present (Supplementary Fig. 15b). The peptides significantly covered lipid A tails as the SASA was reduced dramatically from 100 nm^2 to less than 20 nm^2 over the course of the simulations. The SASA of the lipid headgroup also decreased from 220

nm^2 to 160 nm^2. Contact analysis revealed that hydrophobic residues such as F3, L5, W8, and I9 made most contacts with the lipid acyl chains (Supplementary Fig. 15c). While interactions with the polar headgroups of the lipids span almost all residues of the peptide, basic residues, such as H1, R4, K7 and K11, made prominent contacts with the phosphate moieties. Some of these residues form the KKWIQK sequence of the evolutionarily conserved TCP innate fold known to interact with LPS. Apart from direct interactions with lipid A, the sHVF18 peptide also perturbed the cross-links between lipid A headgroups by sequestering the Ca^{2+} ions. The number of ions interacting with the phosphate groups of lipid A was notably lower than in simulations without the peptide (Supplementary Fig. 15d). The negatively charged C-terminal E18 residue attracted the Ca^{2+} ions away from lipid A (Supplementary Fig. 15e).

We also repeated our CG simulations with *E. coli* rough Ra LPS containing lipid A with ten additional core sugars present in the headgroup. Similarly, sHVF18 became adsorbed onto the surface of an LPS aggregate, primarily in the lipid A region (Supplementary Fig. 16a), highlighting the importance of the lipid A component of LPS in this interaction. As previously described, the adsorption of peptides onto the LPS aggregate significantly reduced the SASA of the lipid tails (Supplementary Fig. 16b), which was driven by interactions with hydrophobic residues of the peptides (Supplementary Fig. 16c). Similarly, Ca^{2+} ions bound to the headgroup were displaced (Supplementary Fig. 16d) due to the interaction of the phosphates with the C-terminal E18 residue of the peptide (Supplementary Fig. 16e).

To further verify our CG MD simulations, we performed atomistic MD simulations whereby one sHVF18 peptide was placed nearby to a small lipid A micelle. As expected, within the first 100 ns, the peptide bound to the surface of the lipid aggregate. Interestingly, the peptide retained most of the secondary structure obtained from NMR, with two α-helices separated by the staple (Supplementary Fig. 18a-c). This suggests that our TFE-derived NMR structure of sHVF18 could represent a physiologically relevant LPS-bound state. Clustering and contact analyses revealed a similar mode of interaction between sHVF18 and lipid A as observed in the CG simulations (Supplementary Fig. 18d and e). The N-terminal basic residues (H1 and R4) of the peptide formed salt bridges with the phosphate groups of lipid A, while the C-terminal acidic residue (E18) formed ionic interactions with the Ca^{2+} ions. Hydrophobic residues along the peptide (F3, L5, I9, and F16) formed hydrophobic interactions with the acyl chains of the lipids. Our atomistic simulations thus corroborate the ability of the stapled peptide to strongly bind to lipid A and encapsulate it, resembling results for linear counterparts as described in our previous study [2].

Supplementary Tables

Supplementary Table 1. Summary of all the peptides detected by LC-MS/MS after digestion of linear and stapled GKY25 with human neutrophil elastase (HNE) and *Pseudomonas* elastase (PE) for 30 min and 3 h.

Sequence	GKY25				sGKY25			
	HNE		PE		HNE		PE	
	30 min	3 h	30 min	3 h	30 min	3 h	30 min	3 h
GKYGFYTHVFRLKKWIQKVIDQFGE	2							
GKYGFYTHVFRLK		2						
GKYGFYTHVFR			4					
GKYGFYT	3	4						
KYGFYTHVFRLKKWIQKV	4	4						
KYGFYTHV	4	4						
YGFYTHVFRLKKWIQKVI	2							
YGFYTHVFRLKKWI	2	3						
YGFYTHVFRL	3	3						
YGFYTHV	4	4						
GFYTHVFRLKKWIQKVI	2							
GFYTHVFRLKKWI	2	3						
GFYTHVFRL	4							
FYTHVFRLKKWIQKV		2						
FYTHVFRL		2						
YTHVFRLKKWIQKV	4	4						
YTHVFRLKKWI	2	2						
YTHVFRL	4	4						
THVFRLKKWIQKVI								
THVFRLKKWIQKV	2	4						
THVFRLKK			3	4				
HVFRLKKWIQKVIDQFGE	2							
VFRLKKWIQKV								
VFRLKKW			2	3				
FRLKKWIQKVIDQFGE	2							
FRLKKWIQK		3						
FRLKKWI	2	2						
FRLKKW		2						
LKKWIQKVIDQFGE			2					
WIQKVIDQFGE				2				
WIQKVIDQ				3				
RLKKWIQKV		2						
KKWIQKVIDQFGE	4							
KKWIQKVI	2	2						
KKWIQKV	2	4						
IQKVIDQFGE			4	4				
QKVIDQFGE	3	2						
KVIDQFGE		4						

Supplementary Table 2. Summary of all the peptides detected by LC-MS/MS after digestion of linear and stapled HVF18 with human neutrophil elastase (HNE) and *Pseudomonas* elastase (PE) for 30 min and 3 h.

	HVF18				sHVF18			
Sequence	HNE		PE		HNE		PE	
	30 min	3 h	30 min	3 h	30 min	3 h	30 min	3 h
HVFRLKK			2	2				
VFRLKKW				1				
VFRLKKWIQKVIDQFGE					1			
FRLKKWI	1							
FRLKKWIQKV	1	3						
LKKWIQKVIDQFGE							2	
KKWIQKV	2	2						
KKWIQKVI	2	1						
KKWIQKVIDQFGE	2				1			
WIQKVIDQ				1				
WIQKVIDQFGE			2					2
IQKVIDQ			2	2				
IQKVIDQFGE	3			2				
QKVIDQFGE	2	2						
KVIDQFGE	2	2		1				
VIDQFGE	1		1	1				

Supplementary Table 3. Assigned chemical shifts of sHVF18.

Residue number	Residue type	H	HA	HB	HG	HD	HE	HZ	HH	CG	CD	CE	CZ	CH
1	His	-	3.98	1.78<br>2.06	-	7.48	7.3	-	-	-	135	-	-	-
2	Val	7.22	3.92	2	0.74<br>0.74	-	-	-	-	19.22<br>19.22	-	-	-	-
3	Phe	8.23	3.49	2.68<br>2.85	-	6.89	6.6	6.7	-	-	-	-	-	-
4	Arg	8.24	4.45	1.67<br>1.78	1.44<br>1.52	3.07<br>3.07	-	-	-	-	-	-	-	-
5	Leu	8.25	4.18	1.58<br>1.63	0.84	0.85<br>0.91	-	-	-	-	22.66<br>22.43	-	-	-
6	Lys	-	-	-	-	-	-	-	-	-	-	-	-	-
7	Lys	-	-	-	-	-	-	-	-	-	-	-	-	-
8	Trp	-	4.2	2.59<br>3.17	-	7.17	10.90<br>7.29	7.46<br>7.02	6.9	-	124	112	121.36<br>118.06	118.6
9	Ile	7.73	3.51	2.1	0.80<br>1.05<br>1.05	0.8	-	-	-	15.2	11.2	-	-	-
10	Lys	-	4	-	1.39<br>1.45	1.57<br>1.67	-	-	-	-	-	-	-	-
11	S5	-	-	-	-		-		-	-	-	-	-	-
12	Val	7.44	3.64	1.9	0.76<br>0.76	-	-	-	-	14.93<br>14.93				-
13	Ile	7.42	3.65	1.9	1.54<br>1.33<br>1.15	0.67	-	-	-	19.6	9.71	-	-	-
14	S5	-	-	-	-	-	-	-	-	-	-	-	-	-
15	Gln	-	2.66	1.33<br>1.33	1.47<br>1.47	-	6.67<br>6.92	-	-	-	-	-	-	-
16	Phe	7.83	4.34	3.16<br>2.93	1.86<br>1.95	7.42	7.19<br>6.92	7.1	-	-	-	-	-	-
17	Gly	7.45	3.76<br>3.66	-	-	-	-	-	-	-	-	-	-	-
18	Glu	7.52	3.77	1.6	1.95	-	-	-	-	-	-	-	-	-

Supplementary Table 4. ^{1}H shifts of sHVF18 in 50% TFE.

Resnr	Resid	H	HA	HB	HG	HD	HE	HZ	HH
1	H	-	4.39	3.37,3.37	-	7.27	8.47	-	-
2	V	7.82	4.33	2.24	1.10,1.08	-	-	-	-
3	F	-	4.43	3.48,3.31	-	7.46	7.59	7.39	-
4	R	8.55	4.81	1.93,1.93	1.62,1.62	3.31,3.31	-	-	-
5	L	7.81	4.43	1.78,1.84	1.82	1.14,1.08	-	-	-
6	K	8.24	4.45	1.98,2.05	1.85,1.79	-	3.38,3.38	-	-
7	K	8.06	4.21	2.03,2.15	1.57,1.63	1.87,1.87	3.12,3.12	-	-
8	W	7.85	4.62	3.62,3.54	-	7.4	7.60,9.95	7.36,7.75	7.26
9	I	7.92	3.83	1.96	1.29,1.70, 1.06	0.94	-	-	-
10	S1	8.7	1.61	2.11,2.19	1.63,1.58	2.25,1.76	5.63	-	-
11	K	7.99	4.28	1.93,1.93	1.62,1.75	1.92,1.62	3.18,3.18	-	-
12	V	7.93	3.77	2.46	1.02,1.10	-	-	-	-
13	I	7.79	3.82	2.24	1.41,1.81,1.05	1.03	-	-	-
14	S1	7.91	1.61	2.12,1.70	1.31,1.80	2.22,1.80	5.5	-	-
15	Q	7.85	4.19	2.19,2.34	2.02,2.19	-	6.63, 7.17	-	-
16	F	8.41	4.83	3.49,3.32	-	7.59	7.46	7.37	-
17	G	8.72	4.19,4.03	-	-	-	-	-	-
18	E	7.89	4.47	2.35,2.19	2.60,2.60	-	-	-	-

Supplementary Table 5. ^{15}N and ^{13}C shifts of sHVF18 in 50% TFE.

Resnr	Resid	N	NE	CA	CB	CG	CD	CE	CZ	CH
1	H	-	-	55.8	30.7	-	120.4	137.5	-	-
2	V	115.5	-	63.0	33.0	20.84,20.86	-	-	-	-
3	F	-	-	56.9	39.5	-	131.6	132.1	129.7	-
4	R	123.9	-	59.4	32.8	25.5	39.5	-	-	-
5	L	121.3	-	56.8	42.6	27.2	23.90,23.92	-	-	-
6	K	122.5	-	57.0	30.8	27.5	-	43.5	-	-
7	K	118.1	-	59.1	32.7	25.4	29.6	42.2	-	-
8	W	118.3	127.3	59.7	29.1	-	126.5	114.4	120.80, 124.67	122.0
9	I	118.8	-	64.2	38.4	16.82, 28.6	10.8	-	-	-
10	S1	127.5	-	20.9	40.6	26.7	30.4	131.2	-	-
11	K	118.6	-	58.8	32.8	25.5	29.6	42.3	-	-
12	V	120.1	-	66.7	31.8	20.37,21.97	-	-	-	-
13	I	119.2	-	65.4	36.7	16.59,28.27	12.6	-	-	-
14	K	131.8	-	22.6	41.7	26.1	29.7	132.9	-	-
15	Q	113.9	109.3	58.6	34.3	29.3	-	-	-	-
16	F	117.9	-	58.5	39.5	-	131.6	132.1	129.9	-
17	G	109.4	-	45.7	-	-	-	-	-	-
18	E	123.0	-	57.0	30.0	34.0	-	-	-	-

Supplementary Table 6. Summary of conformationally-restricting experimental constraints.

	Protein
NMR distance and dihedral constraints	
Distance constraints	
Total NOE	389
Intra-residue	188
Inter-residue	
Sequential ($\|i-j\| = 1$)	98
Medium-range ($\|i-j\| < 4$)	98
Long-range ($\|i-j\| > 5$)	5
Intermolecular	
Hydrogen bonds	
Total dihedral angle restraints	
ϕ	13
ψ	13
Structure statistics	
Violations (mean and s.d.)	
Distance constraints (Å)	0.0092±0.074
Dihedral angle constraints (º)	1.82±2.20
Max. dihedral angle violation (º)	53.6
Max. distance constraint violation (Å)	0
Average pairwise r.m.s. deviation** (Å)	
Heavy	5.02
Backbone	1.33

Supplementary Table 7. Residue-specific RMSD values for backbone and all atoms and average backbone dihedral angles for sHVF18.

Residue N.	Residue Type	Backbone RMSD	All atom RMSD	Phi angle	Psi angle
1	His	8.7	9.6	None	None
2	Val	7.1	7.5	-116.9	16.1
3	Phe	6.0	7.7	-125.3	-25.9
4	Arg	4.6	6.6	-103.1	-3.0
5	Leu	2.9	3.9	-120.9	156.1
6	Lys	2.7	4.4	-72.1	17.5
7	Lys	2.3	4.4	-134.9	-13.4
8	Trp	1.6	3.0	-85.5	1.0
9	Ile	1.0	1.2	None	None
10	S5	N/A	N/A	None	None
11	Lys	0.7	1.4	None	None
12	Val	0.4	0.8	-63.1	-51.2
13	Ile	0.9	1.5	None	None
14	S5	N/A	N/A	None	None
15	Gln	0.7	1.2	None	None
16	Phe	1.2	2.7	-87.2	39.3
17	Gly	2.5	2.7	-137.0	20.8
18	Glu	4.4	5.7	None	None

Supplementary Table 8. List of simulations performed in this study.

System	Forcefield	Box dimensions (nm)	Number of atoms		Salt concentration	Simulation length (µs)
			Total	Water		
CD14-sHVF18 model 1	CHARMM36m all-atom	11 x 11 x11	114521	36390	0.15 M NaCl	1 x 0.2
CD14-sHVF18 model 2	CHARMM36m all-atom	11 x 11 x 11	112097	35584	0.15 M NaCl	1 x 0.2
CD14-sHVF18 model 3	CHARMM36m all-atom	10 x 10 x 10	91063	28586	0.15 M NaCl	1 x 0.2
CD14-sHVF18 model 4	CHARMM36m all-atom	10 x 10 x 10	93760	29483	0.15 M NaCl	1 x 0.2
CD14-sHVF18 model 5	CHARMM36m all-atom	10 x 10 x 10	93652	29447	0.15 M NaCl	1 x 0.2
CD14-sHVF18 model 6	CHARMM36m all-atom	10 x 10 x 10	97109	30598	0.15 M NaCl	1 x 0.2
CD14-sHVF18 model 7	CHARMM36m all-atom	10 x 10 x 10	105356	33341	0.15 M NaCl	1 x 0.2
CD14-sHVF18 model 8	CHARMM36m all-atom	10 x 10 x 10	102560	32411	0.15 M NaCl	1 x 0.2
CD14-sHVF18 model 9	CHARMM36m all-atom	10 x 10 x 10	97208	30631	0.15 M NaCl	1 x 0.2
CD14-sHVF18 model 10	CHARMM36m all-atom	10 x 10 x 10	102497	32390	0.15 M NaCl	1 x 0.2
CD14-sHVF18 model 11	CHARMM36m all-atom	10 x 10 x 10	102548	32407	0.15 M NaCl	1 x 0.2
CD14-sHVF18 top cluster	CHARMM36m all-atom	10 x 10 x 10	93452	29381	0.15 M NaCl	3 x 1
Apo CD14	CHARMM36m all-atom	10 x 10 x 10	93637	29555	0.15 M NaCl	3 x 1
Lipid A with sHVF18	Martini 2.2 coarse-grained	14 x 14 x 14	23920	19820	0.15 M NaCl + ~0.07 M Ca^{2+}	3 x 10
Lipid A control	Martini 2.2 coarse-grained	14 x 14 x 14	22897	20327	0.15 M NaCl + ~0.07 M Ca^{2+}	3 x 10
Ra LPS with sHVF18	Martini 2.2 coarse-grained	17 x 17 x 17	42924	35946	0.15 M NaCl + ~0.08 M Ca^{2+}	3 x 10
Ra LPS control	Martini 2.2 coarse-grained	15 x 15 x 15	28635	23485	0.15 M NaCl + ~0.08 M Ca^{2+}	3 x 10
Lipid A with sHVF18 validation	CHARMM36m all-atom	10 x 10 x 10	85342	27075	0.15 M NaCl + ~0.05 M Ca^{2+}	3 x 1

Supplementary figures

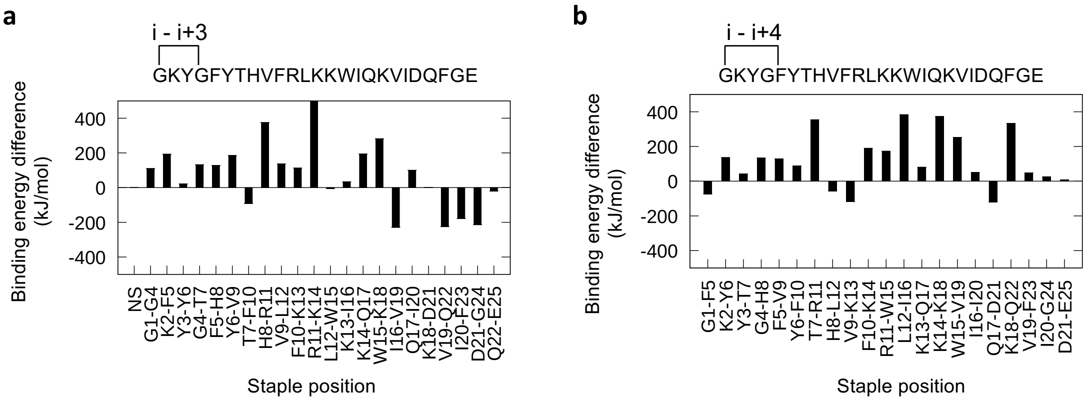

Supplementary Fig. 1 | *In silico* analysis of staple positions. a, HVF18 was docked onto CD14, and the N-terminal GKYGFYT residues were modeled in the GKY25 peptide. The binding energy of GKY25 to CD14 was calculated using MMPBSA. A pentenyl alanine staple was added to consecutive connecting residues i and i+3 along the sequence of the peptide. MMPBSA was used to calculate the binding energy of the stapled peptide to CD14. The graph shows the binding energy difference between non-stapled and stapled GKY25 for all staple positions. Positive values indicate worse binding, while negative values indicate more favorable binding for the stapled peptide. **b**, A similar analysis was performed by adding the staple to consecutive connecting residues i and i+4 along the sequence of the peptide.

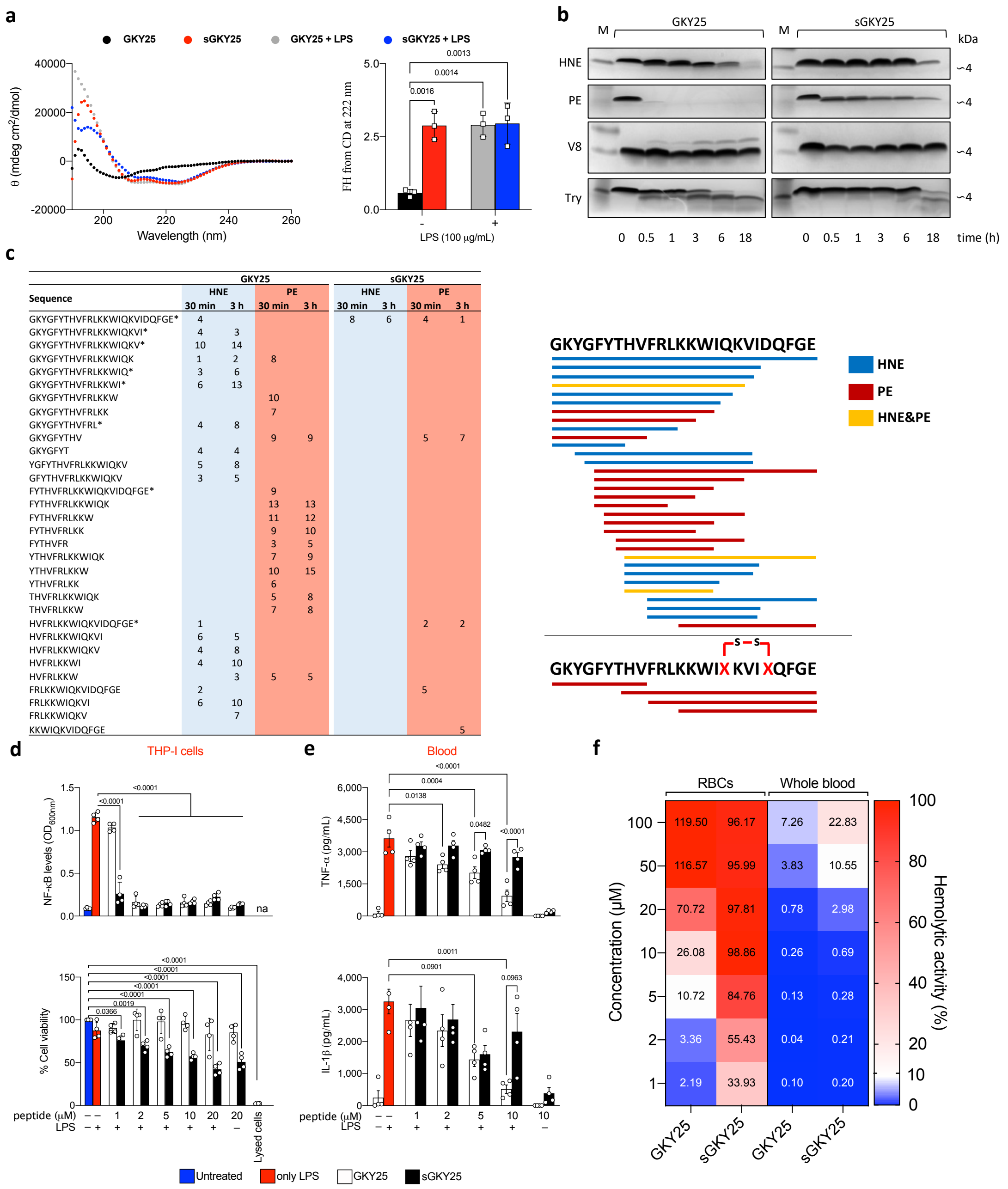

Sequence	GKY25 HNE 30 min	GKY25 HNE 3 h	GKY25 PE 30 min	GKY25 PE 3 h	sGKY25 HNE 30 min	sGKY25 HNE 3 h	sGKY25 PE 30 min	sGKY25 PE 3 h
GKYGFYTHVFRLKKWIQKVIDQFGE*	4				8	6	4	1
GKYGFYTHVFRLKKWIQKVI*	4	3						
GKYGFYTHVFRLKKWIQKV*	10	14						
GKYGFYTHVFRLKKWIQK	1	2	8					
GKYGFYTHVFRLKKWIQ*	3	6						
GKYGFYTHVFRLKKWI*	6	13						
GKYGFYTHVFRLKKW			10					
GKYGFYTHVFRLKK			7					
GKYGFYTHVFRL*	4	8						
GKYGFYTHV			9	9			5	7
GKYGFYT	4	4						
YGFYTHVFRLKKWIQKV	5	8						
GFYTHVFRLKKWIQKV	3	5						
FYTHVFRLKKWIQKVIDQFGE*			9					
FYTHVFRLKKWIQK			13	13				
FYTHVFRLKKW			11	12				
FYTHVFRLKK			9	10				
FYTHVFR			3	5				
YTHVFRLKKWIQK			7	9				
YTHVFRLKKW			10	15				
YTHVFRLKK			6					
THVFRLKKWIQK			5	8				
THVFRLKKW			7	8				
HVFRLKKWIQKVIDQFGE*	1						2	2
HVFRLKKWIQKVI	6	5						
HVFRLKKWIQKV	4	8						
HVFRLKKWI	4	10						
HVFRLKKW		3	5	5				
FRLKKWIQKVIDQFGE	2						5	
FRLKKWIQKVI	6	10						
FRLKKWIQKV		7						
KKWIQKVIDQFGE								5

Supplementary Fig. 2 | Biophysical and biochemical analysis of stapled GKY25. a, Representative CD spectra of linear and stapled GKY25 in the presence and absence of LPS. The experiment was performed three times (n=3). The relative α-helical content at 222 nm was calculated from CD spectra. The data are presented as mean ± SEM. Significance was established by an ordinary two-way ANOVA followed by Tukey's multiple comparisons tests using GraphPad Prism software. **b**, SDS-PAGE of intact and digested peptides with different proteases for different lengths of time. One representative image from three independent experiments is shown (n=3). **c**, Table and graphical representation summarize all the major peptides obtained after digestion with human neutrophil elastase (HNE) and *Pseudomonas* elastase (PE) for 30 min and 3 h, using mass spectrometry. Asterisks (*) indicate peptides

with antibacterial activity. Experiments were performed two times in duplicate, using two different digestions (n=2). **d**, NF-κB activation and cell viability in THP1-XBlue-CD14 reporter cells stimulated with 100 ng ml^{-1} *E. coli* LPS in the presence or absence of increasing concentrations of linear and stapled GKY25, 20 h post-stimulation. Results are presented as means ± SD of four experiments (n=4). Significance was determined by an ordinary two-way ANOVA followed by Tukey's multiple comparisons tests using GraphPad Prism software. **e**, Cytokines released from human blood stimulated with 100 ng ml^{-1} *E. coli* LPS in the presence or absence of increasing concentrations of GKY25 and sGKY25, 24 h post-stimulation. Results are presented as mean ± SEM. Blood from a different donor was used each time (n=4). P-values were determined by ordinary two-way ANOVA followed by Tukey's multiple comparisons tests using GraphPad Prism software. **f**, The heatmaps show the hemolytic activity of the peptides on erythrocytes (RBCs) or whole blood. Data are presented as the mean of three independent experiments, each performed with blood from a different donor (n=3).

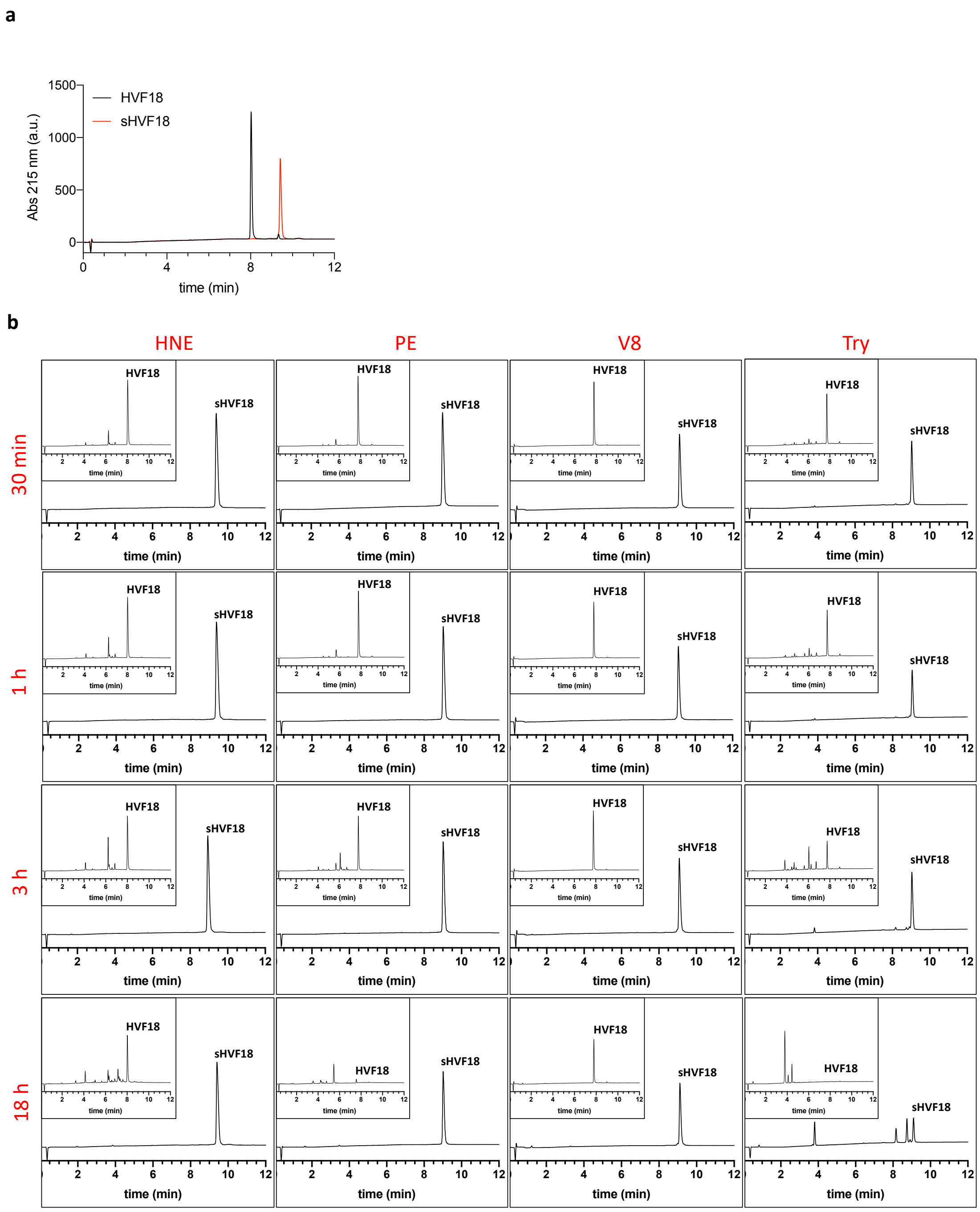

Supplementary Fig. 3 | Evaluation of elution profile of linear and stapled HVF18 on Reverse-phase C18 column. Representative chromatograms of HVF18 and sHVF18 before (**a**) and after digestion with different enzymes (**b**) for different lengths of time (30 min–18 h). NHE, human neutrophil elastase; PE, *P. aeruginosa* elastase; V8, Glutamyl-C endopeptidase from *S. aureus* V8; Try, trypsin.

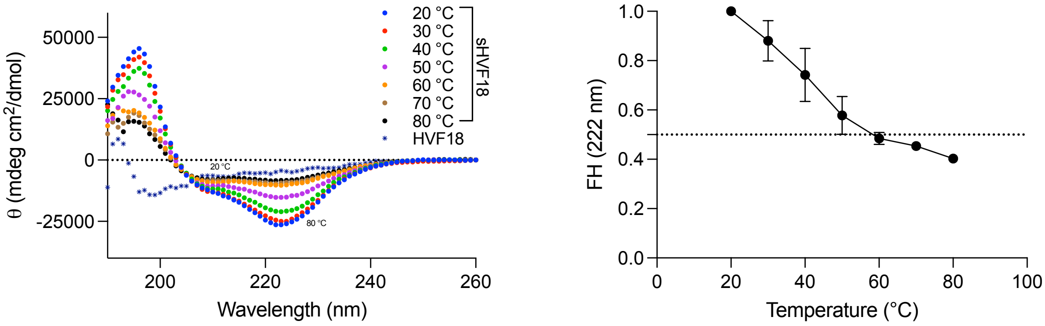

Supplementary Fig. 4 | Thermal denaturation of sHVF18. Representative CD spectra of sHVF18 following incubation at increasing temperatures (20–80°C). The experiment was performed three times (n=3). The relative α-helical content at 222 nm was calculated from CD spectra. The data are presented as mean ± SEM. The spectrum of HVF18 at 20°C was used as a control.

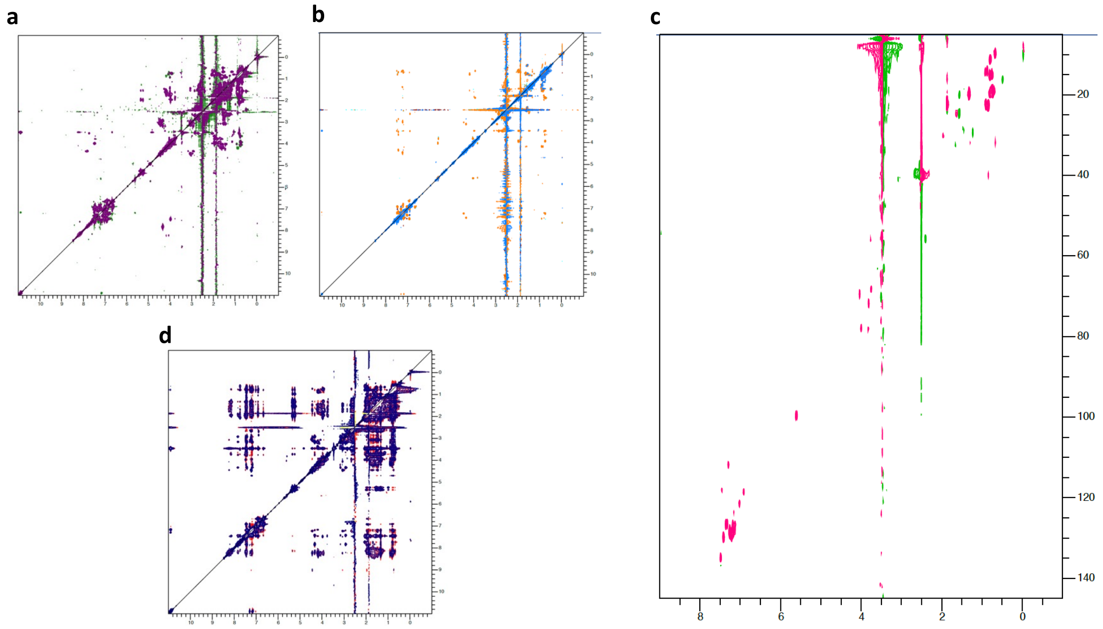

Supplementary Fig. 5 | NMR spectra of sHVF18 in DMSO-d6. a, TOCSY spectra using mixing times of 40 (purple) and 80 ms (green). **b**, ROESY spectra using mixing times of 100 (blue/yellow) and 150 ms (brown/cyan). **c**, 13C-edited-HSQC spectra (green: CH2 spin systems, red: CH or CH3 spin systems). **d**, NOESY spectra of sHVF18 using mixing times of 100 (blue) and 150 ms (red).

a

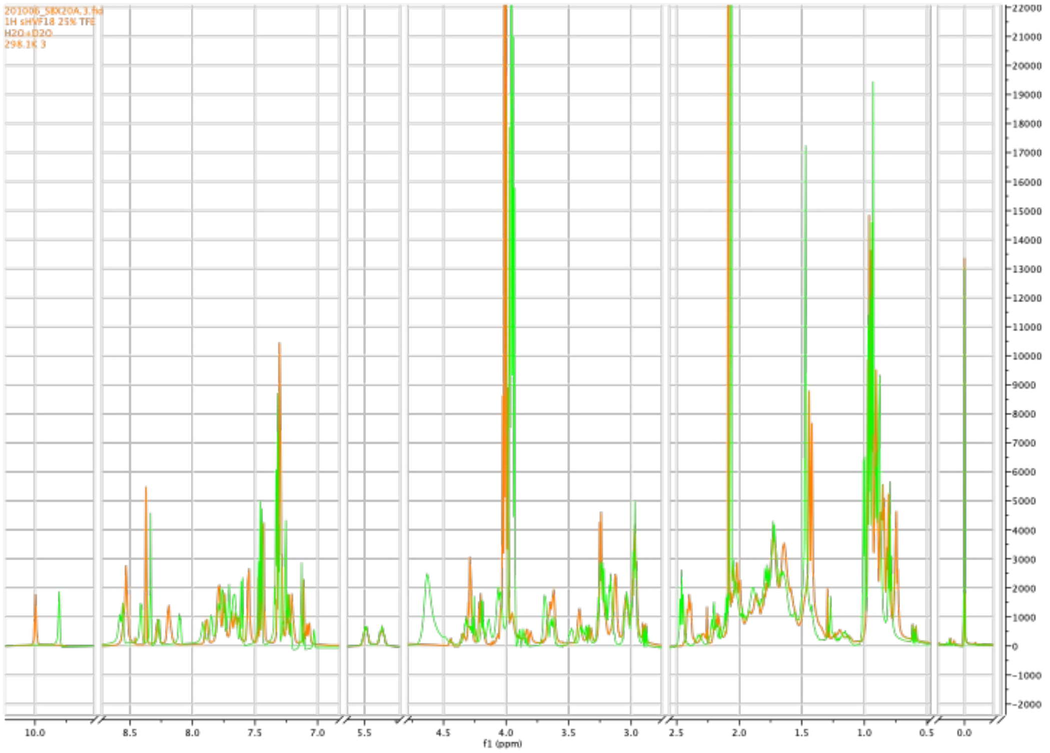

b

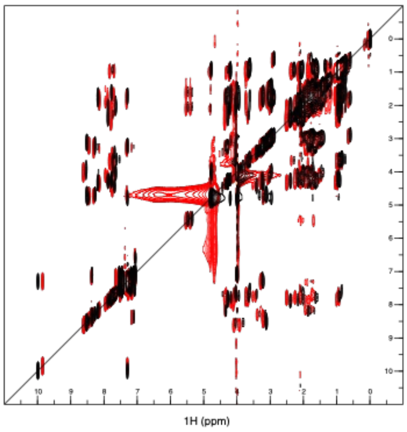

Supplementary Fig. 6 | NMR spectra of sHVF18 in 25 and 50% TFE. a, ^{1}H spectra of sHVF18 in 25% (orange) and 50% TFE (green). **b**, TOCSY spectra (mixing time 80 ms) of sHVF18 in 25% (black) and 50% TFE (red).

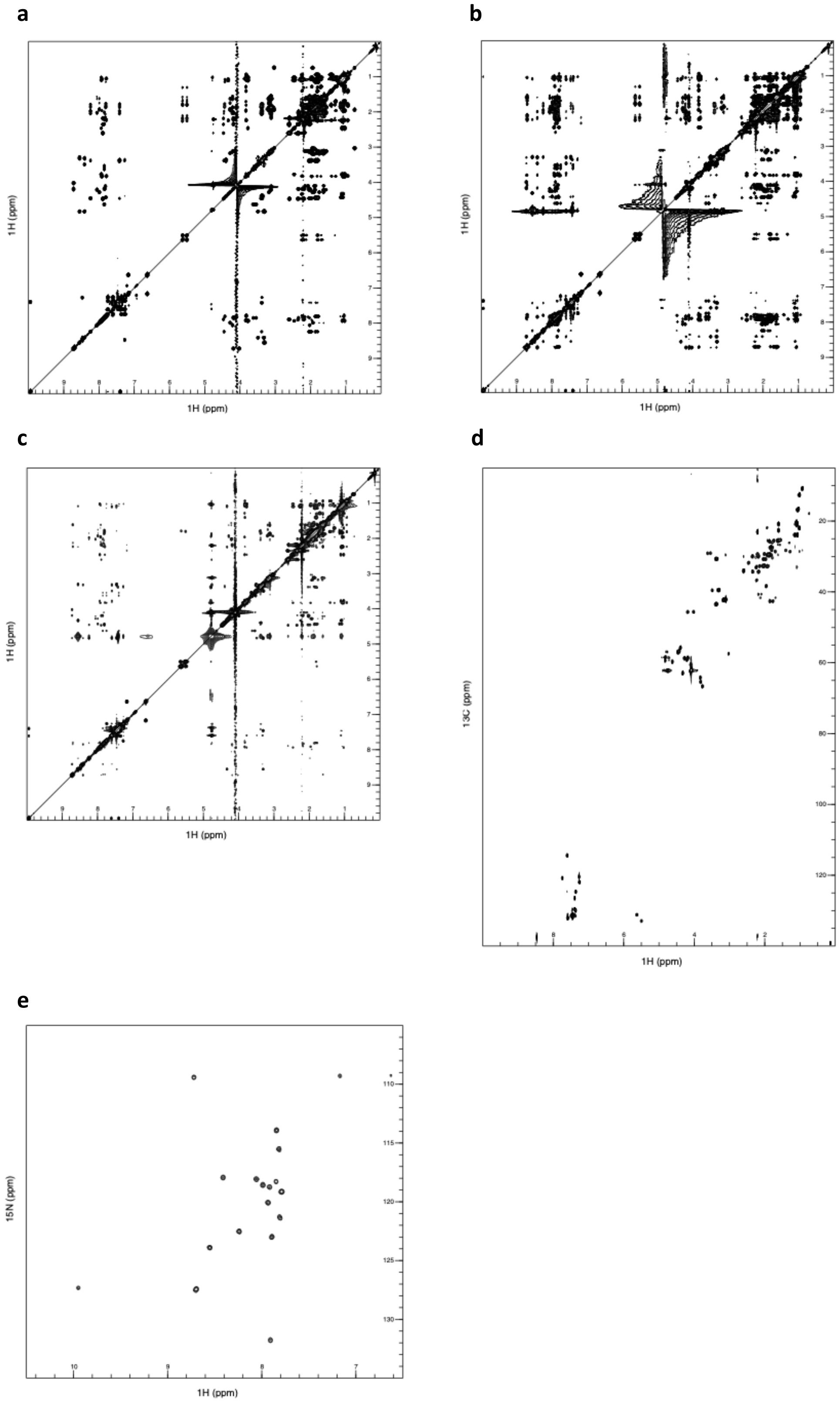

Supplementary Fig. 7 | NMR spectra of sHVF18 in 50% TFE. a, TOCSY, mixing time 80 ms. **b**, NOESY, mixing time 150 ms. **c**, ROESY, mixing time 150 ms. **d**, ^{13}C-HSQC spectrum. **e**, ^{15}N SOFAST-HMQC spectrum .

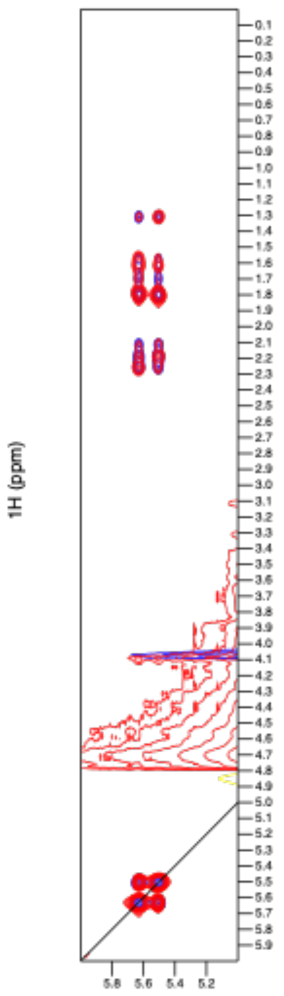

Supplementary Fig. 8 | Cutout of NOESY (red) and TOCSY (blue) spectra for the aromatic peaks for the stapled residues of sHVF18 in 50% TFE.

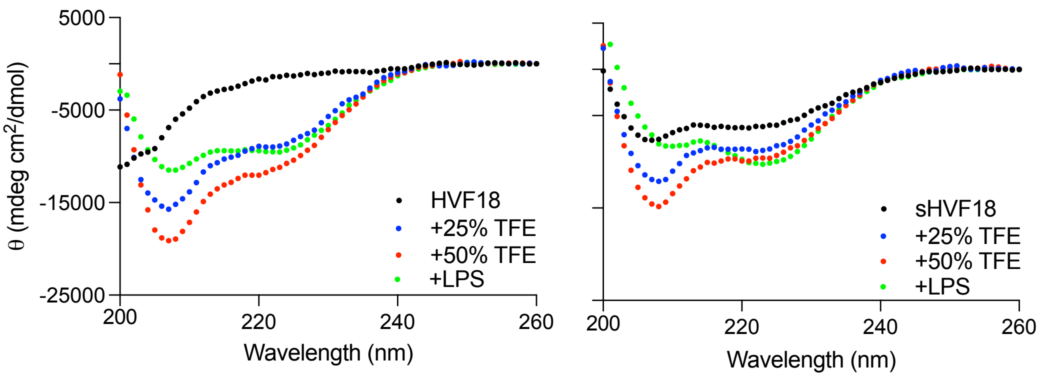

Supplementary Fig. 9 | **Secondary structure of sHVF18 in 25 and 50% TFE.** Representative CD spectra of three independent experiments for HVF18 and sHVF18 diluted in 25 or 50 % TFE or in water with 100 µg mL^{-1} LPS (n=3).

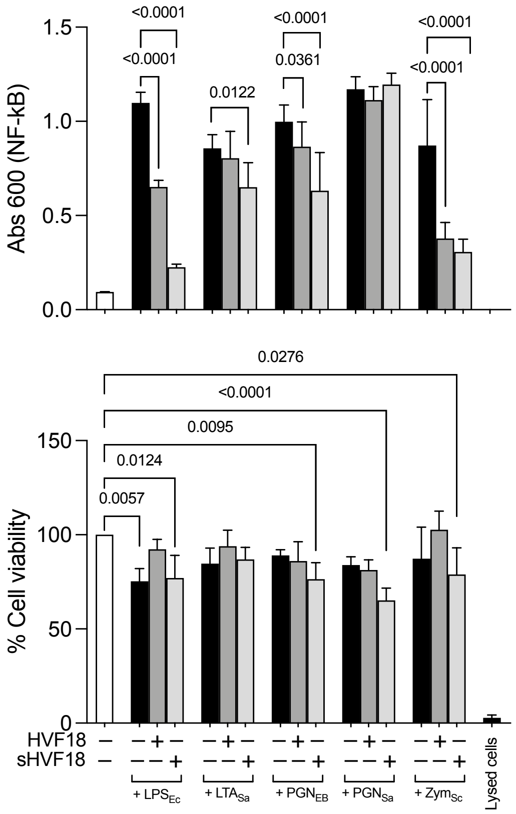

Supplementary Fig. 10 | Anti-inflammatory activity of linear and stapled HVF18. a, NF-kB activation and cell viability in THP1-XBlue-CD14 reporter cells stimulated with 100 ng ml^{-1} of *E. coli* LPS (LPS_{Ec}), 1 µg ml^{-1} *S. aureus* LTA (LTA_{Sa}), 1 µg ml^{-1} *E. coli* PGN (PGN_{EB}), 1 µg ml^{-1} *S. aureus* PGN (PGN_{Sa}), 10 µg ml^{-1} *S. cerevisiae* zymosan (Zym_{Sc}) in the presence or the absence of 10 µM of linear and stapled HVF18 20 h post-stimulation.Results are presented as mean ± SD of four experiments (n=4). P-values were determined using an ordinary two-way ANOVA followed by Tukey´s multiple comparisons tests using GraphPad Prism software.

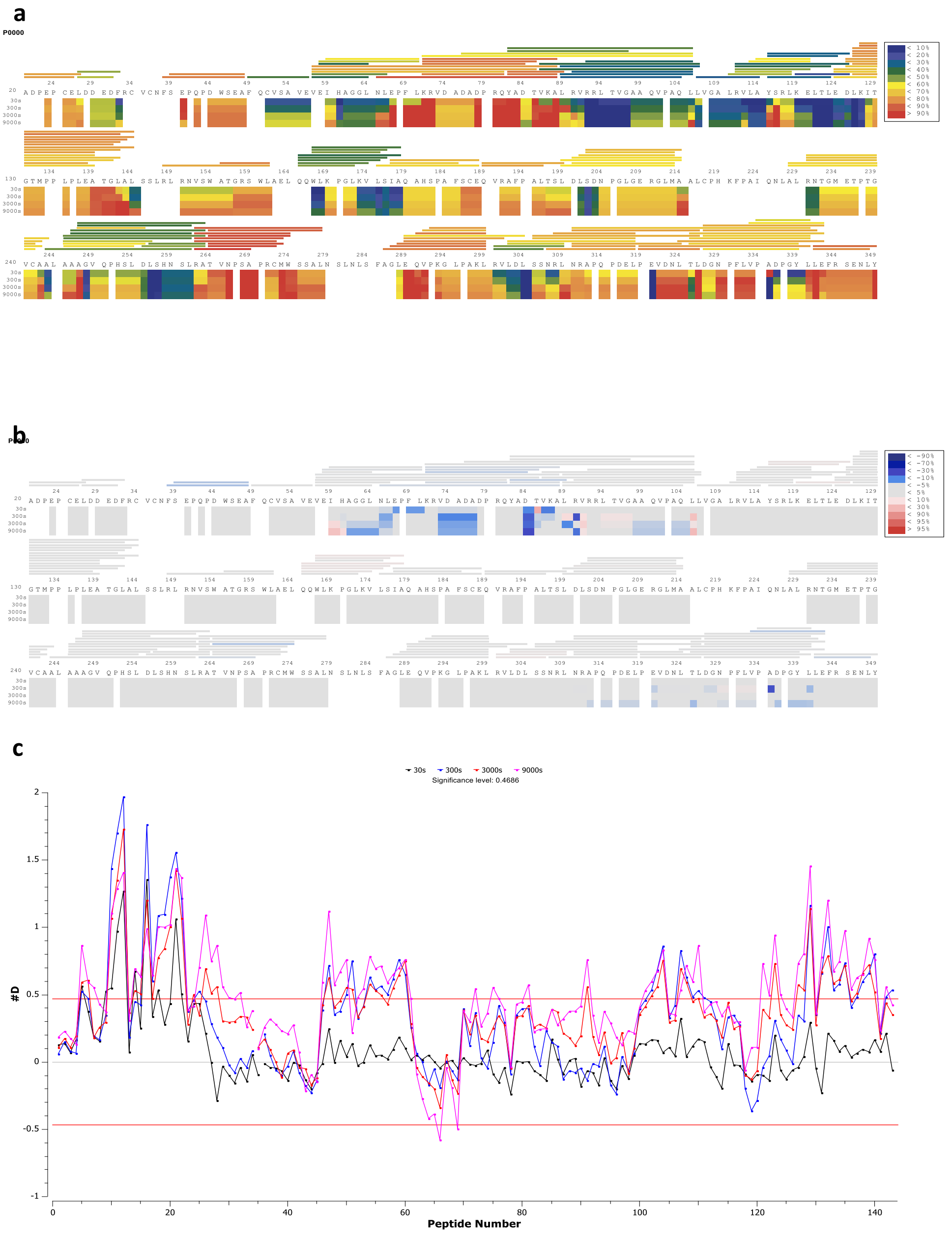

Supplementary Fig. 11 | Binding of LPS to CD14. a, Heatmap for the deuterium uptake of CD14 in the presence of LPS at 30, 300, 3000, and 9000 s time points. The peptide coverage is illustrated by the bars above the heatmap and is color coded to the average deuterium uptake overall observed time points. **b**, The differential heatmap for ligand-free (apo) CD14 and for CD14 with LPS shows the uptake perturbations at the 30, 300, 3000, and 9000 s time points. The differential peptide coverage is illustrated by the bars above the heatmap and is color coded to the average deuterium uptake overall observed time points. **c**, Butterfly plot with a central zero showing the residuals between the apo CD14 state and CD14 with LPS. The different curve colors indicate deuterium uptake at the different time points (top legend). The y-axis is a difference in uptake (Da), and the x-axis is the peptide number.

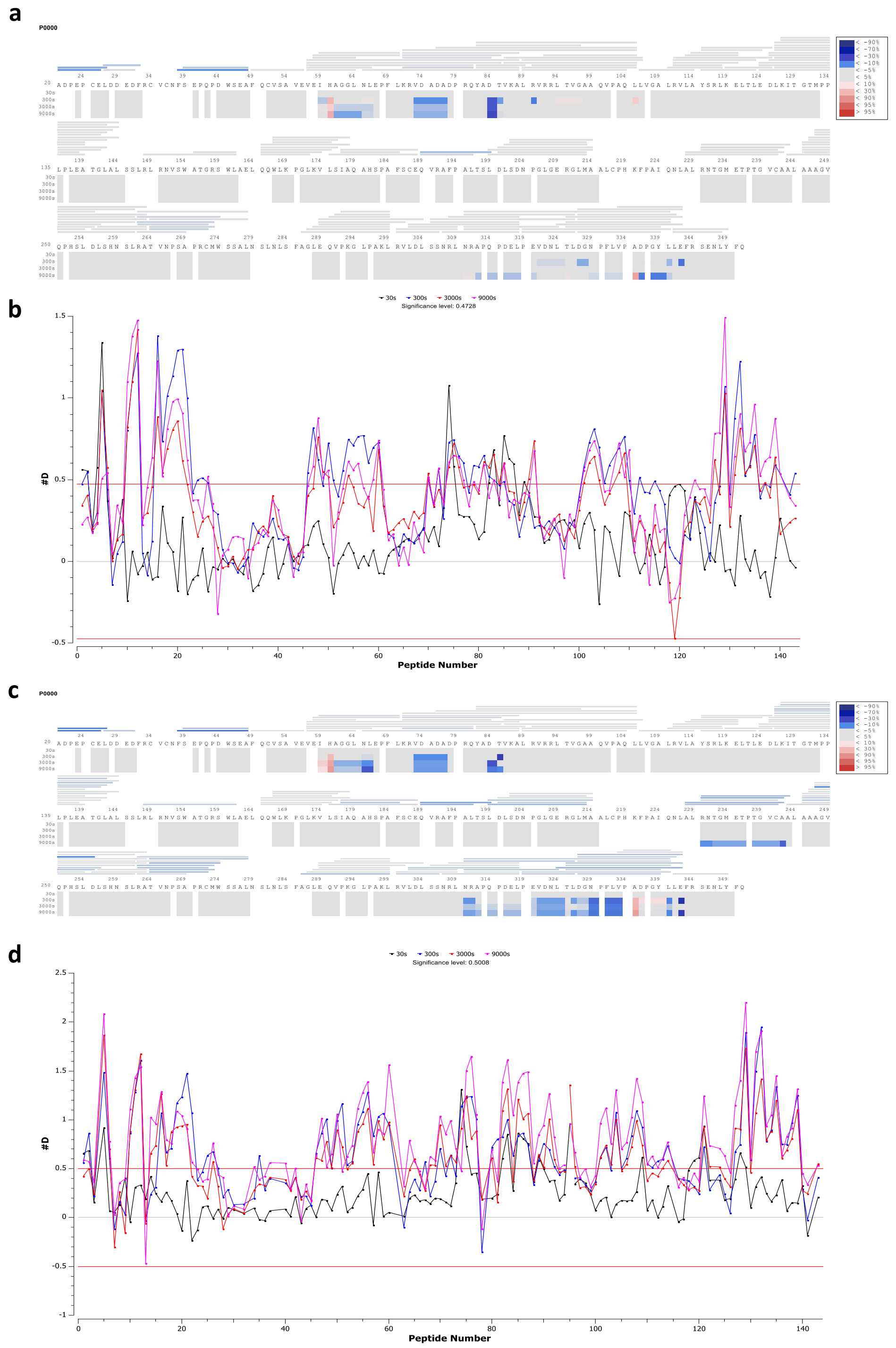

Supplementary Fig. 12 | Binding of linear and stapled HVF18 to CD14. a and **c**, The differential heatmaps of ligand-free (apo) CD14 with sHVF18 (**a**) or HVF18 (**c**) show the uptake perturbations at the 30, 300, 3000, and 9000 s time points. The differential peptide coverage is illustrated by the bars above the heatmap and is color coded to the average deuterium uptake overall observed time points. **b** and **d**, Butterfly plot with a central zero showing the residuals between the apo CD14 state and CD14 with sHVF18 (**b**) or HVF18 (**d**). The different curve colors indicate deuterium uptake at the different time points (top legend). Y-axis is a difference in uptake (Da), and the x-axis is the peptide number.

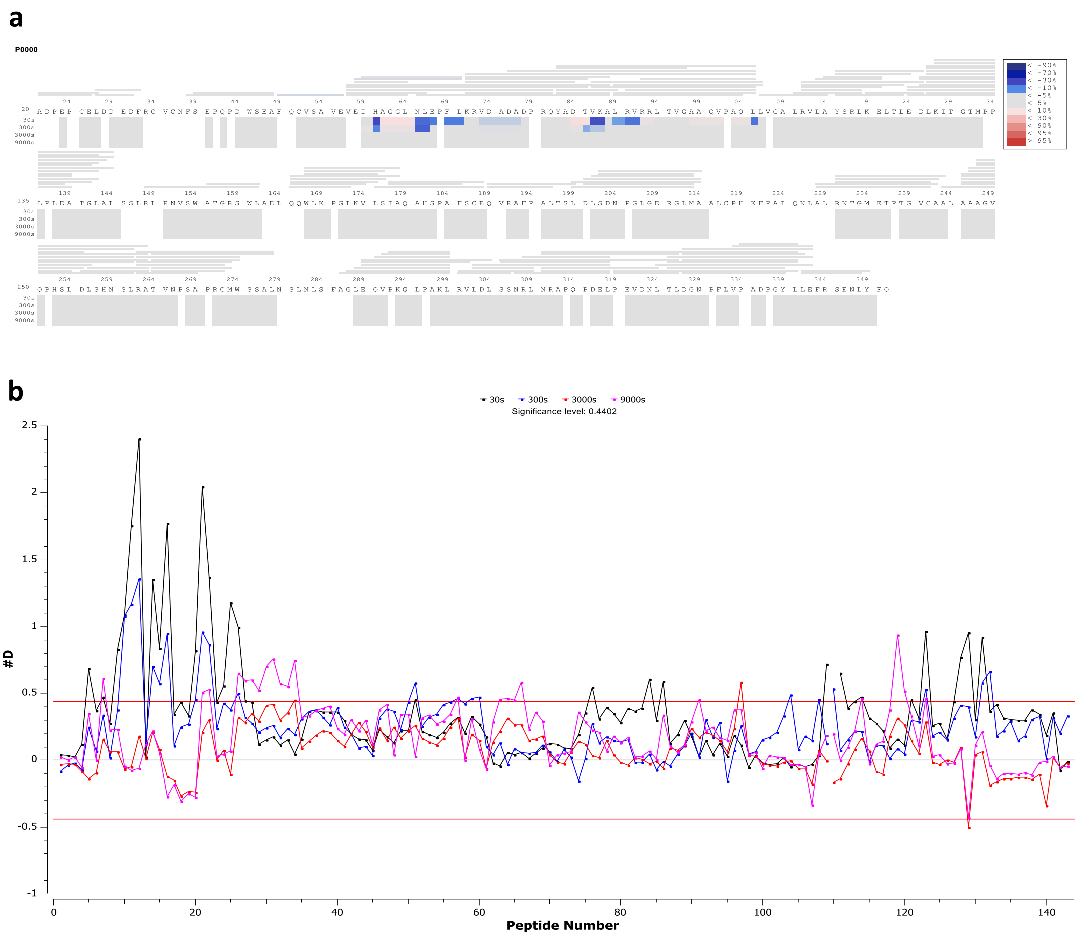

Supplementary Fig. 13 | Binding of sHVF18 to CD14 in the presence of LPS. a, The differential heatmap ligand-free (apo) CD14 with sHVF18+LPS shows the uptake perturbations at the 30, 300, 3000, and 9000 s time points. The differential peptide coverage is illustrated by the bars above the heatmap and is color coded to the average deuterium uptake overall observed time points. **b**, Butterfly plot with a central zero showing the residuals between the CD14 apo state and CD14 with sHVF18+LPS. The different curve colors indicate deuterium uptake at the different time points (top legend). The y-axis is the difference in uptake (Da), and the x-axis is the peptide number.

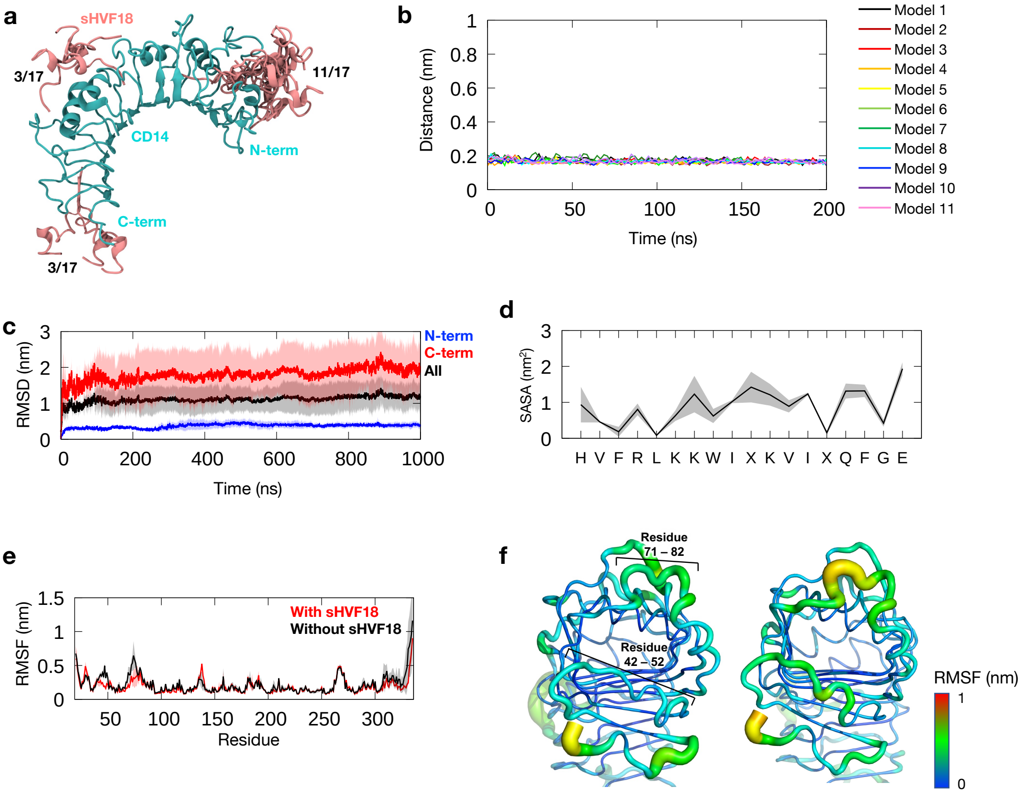

Supplementary Fig. 14 | Docking and MD simulation of sHVF18 peptide with human CD14. a, Unbiased protein-protein docking was performed using the structures of sHVF18 and human CD14 using the ClusPro web server. The figure shows all 17 docking poses generated by ClusPro in cartoon representation, with CD14 in cyan and sHVF18 in pink. The number of docking poses at each binding site is labeled. **b**, Each of the 11 docking poses at the N-terminal binding site were subjected to a 200 ns MD simulation. The figure shows minimum distance between the peptide and the protein throughout the simulations and indicates that the peptide remained bound in all 11 predicted poses. **c**, All 11 simulation trajectories were concatenated and a cluster analysis was performed to extract the most representative structure. The central structure of the top cluster (which comprises around 90% of all trajectories) was subjected to three independent 1 μs simulations. The figure shows the RMSD of backbone atoms of the first five residues at the N-terminus (blue), the last five residues at the C-terminus (red), and all residues (black) after least-squares fitting to the backbone atoms of the CD14-peptide complex. Thick lines show average values from three simulations, and the standard deviations are shown as shaded regions. **d**, Solvent accessible surface area (SASA) of each residue on the sHVF18 peptide during the simulations. A probe radius of 0.14 nm was used. **e**, Per-residue RMSF of CD14 from simulations with and without sHVF18 shown in red and black, respectively. **f**, Average RMSF values projected onto the structure of the protein for simulations with (left) and without sHVF18 (right) focusing on the N-terminal peptide binding site. Regions with significant differences are labeled.

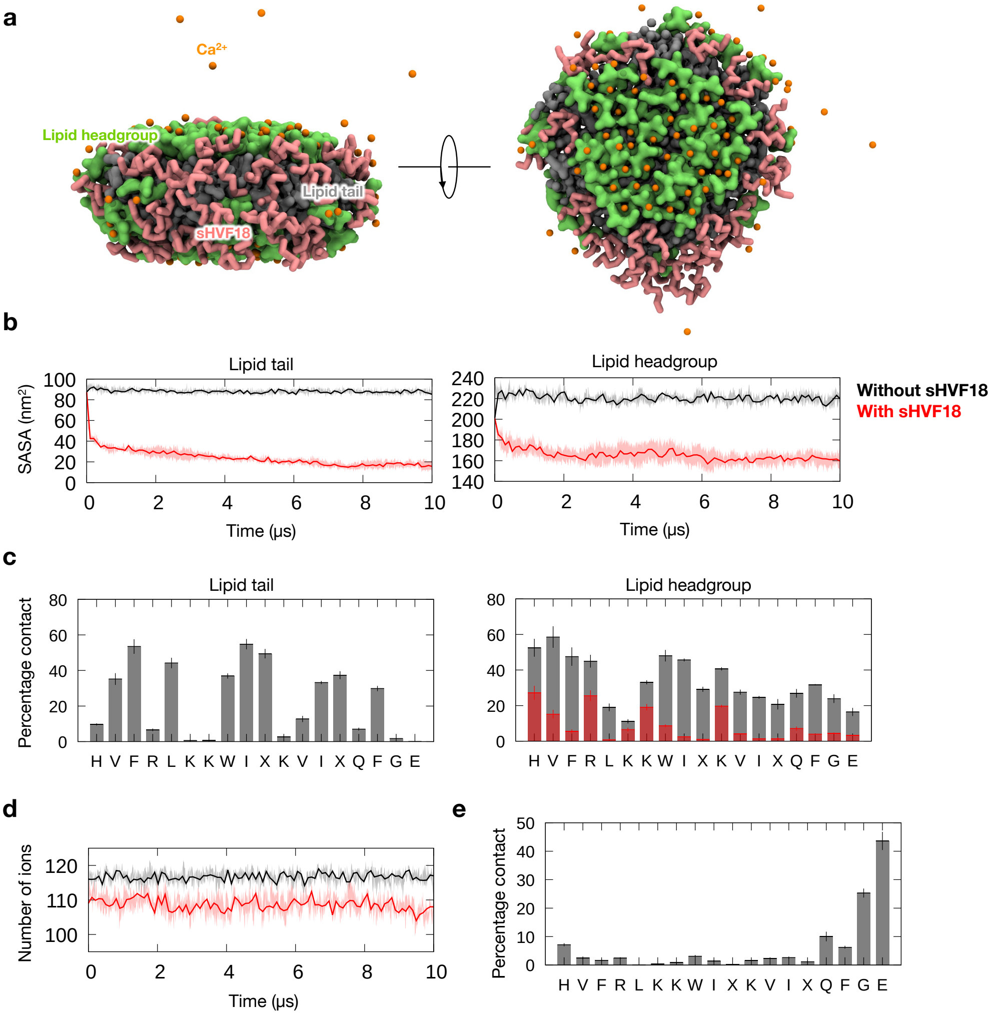

Supplementary Fig. 15 | CG simulations of sHVF18 and lipid A aggregates. a, A lipid A aggregate composed of 60 lipid molecules was simulated with 30 copies of sHVF18 peptides placed at least 2 nm from its surface at the beginning of the simulations. Figures show two snapshots at the end of a 10 μs simulation taken from the side (left) and top (right) of the lipid aggregate. The lipid is shown in surface representation with the headgroups in green and acyl chain tails in grey, while the peptides are shown in stick representation in pink. Only the backbone particles of the peptide are shown for clarity. Ca^{2+} ions are shown as orange spheres. **b**, Solvent accessible surface area (SASA) of the lipid tails (left) and headgroups (right) from simulations with (red) and without sHVF18 peptide (black). A probe radius of 0.26 nm was used for the calculation to represent the radius of water particles in the CG MARTINI force field. Thick lines show averages from three independent 10 μs simulations, and shaded areas show standard deviations. **c**, Percentage of contacts made by each residue of the peptide with lipid tails (left) and headgroups (right). Contacts made with the phosphate particles of the lipid headgroup are highlighted in red. Average values from three simulations are shown, with the standard deviation depicted as error bars. The cut-off distance used for contact measurement is 0.6 nm. **d**, The number of Ca^{2+} ions found within 0.6 nm from the

phosphate particles of lipid A throughout the simulations. **e**, Average percentage of contacts made by each residue of the peptide with Ca^{2+} ions.

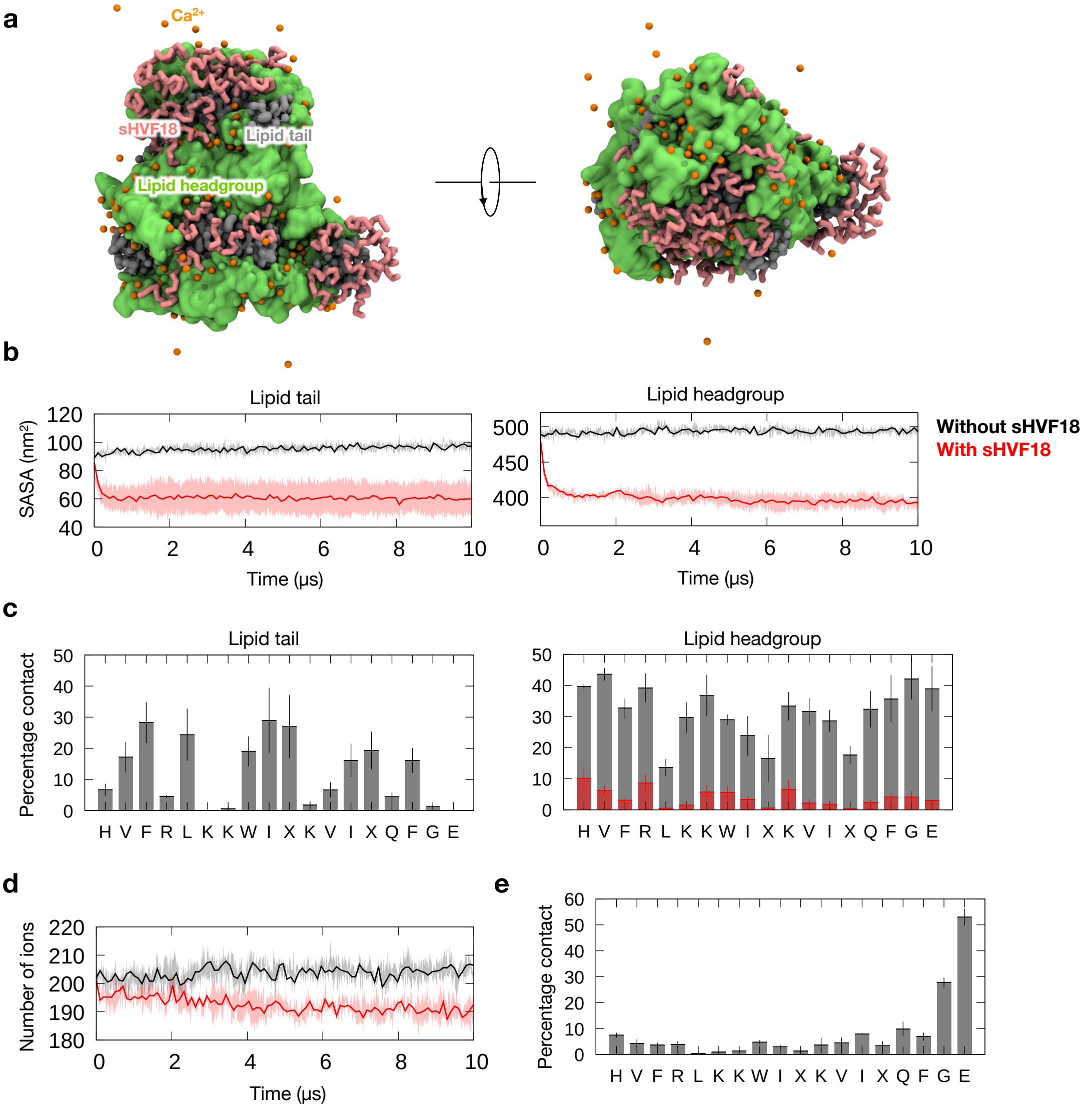

Supplementary Fig. 16 | CG simulations of sHVF18 and LPS aggregate. a, An LPS aggregate was formed by running a 10 µs CG self-assembly simulation of 60 *E. coli* rough Ra LPS molecules (lipid A plus 10 core sugars). Subsequently 30 copies of sHVF18 peptides were added to the system at a minimum distance of 2 nm from the surface of the lipid aggregate. Three replicates of 10 µs simulation were then performed. The figures show two snapshots at the end of one simulation taken from the side (left) and top (right) of the LPS aggregate. LPS is depicted in surface representation with the lipid tails in grey and

headgroups in green. The peptide backbone is shown in stick representation in pink. Ca^{2+} ions are shown as orange spheres. **b**, SASA of the lipid tails (left) and headgroups (right) from simulations with (red) and without sHVF18 peptide (black). A probe radius of 0.26 nm was used for calculation to represent the radius of water particles in CG MARTINI force field. Thick lines show average from three simulations and shaded areas show standard deviations. **c**, Percentage of contact made by each residue of the peptide with lipid tails (left) and headgroups (right). Contacts made with the phosphate particles of the lipid headgroup is highlighted in red. Average values from three simulations are shown with the standard deviation depicted as the error bars. The cut-off distance used for contact measurement is 0.6 nm. **d**, The number of Ca^{2+} ions found within 0.6 nm from the phosphate particles of LPS throughout the simulations. **e**, Average percentage of contact made by each residue of the peptide with Ca^{2+} ions.

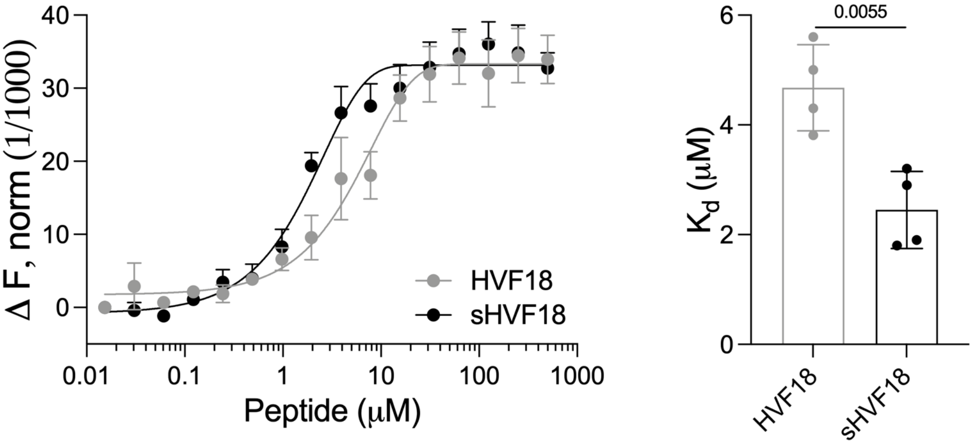

Supplementary Fig. 17 | Affinity of linear and stapled HVF18 to LPS. Representative binding curves between LPS and HVF18 or sHVF18, obtained by MST. K_d values were calculated from MST curves. The data are presented as mean ± SD of four different measurements (n=4). P-values were determined using unpaired t-test analysis using GraphPad Prism software.

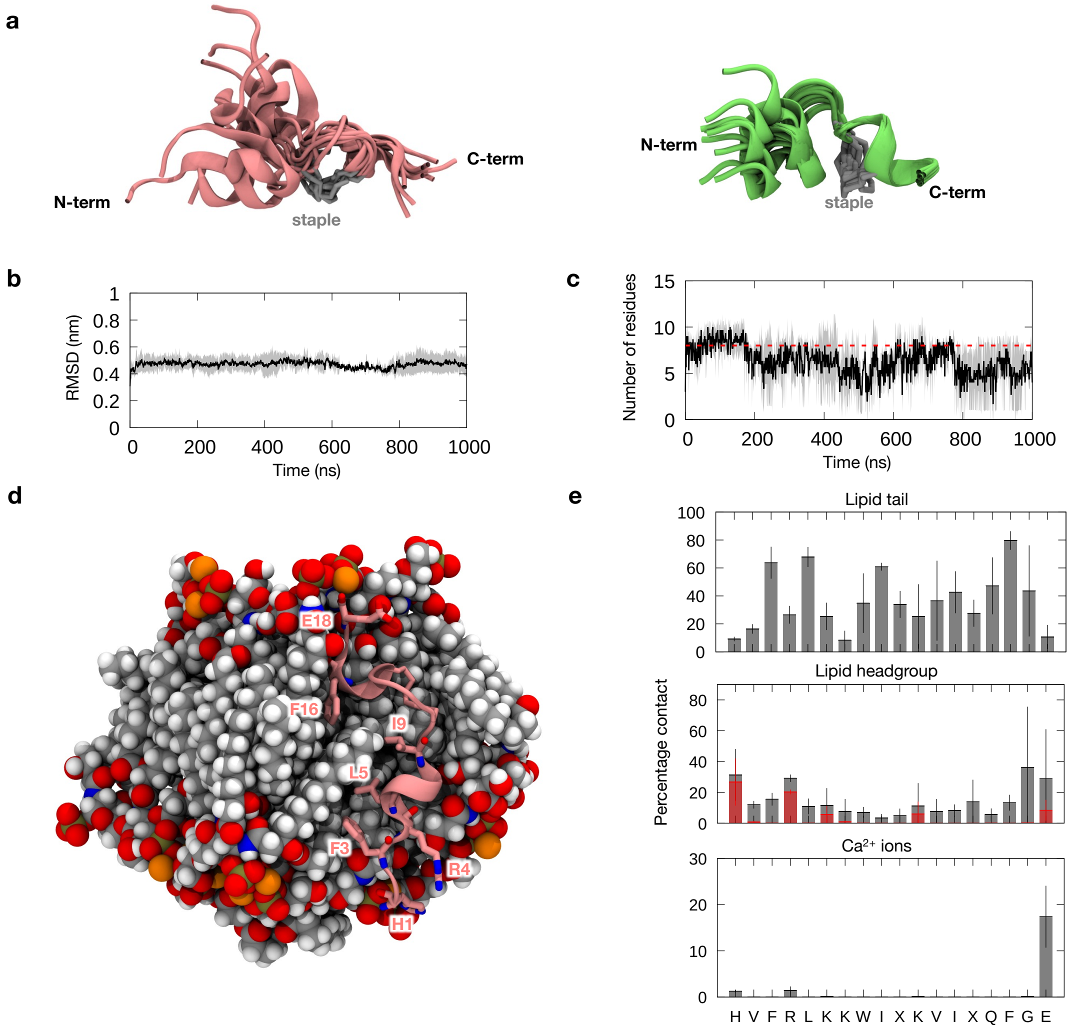

Supplementary Fig. 18 | Atomistic simulations of sHVF18 and a lipid A aggregate. A small lipid A aggregate composed of 12 lipid molecules was simulated with a single sHVF18 peptide placed around 2 nm from its surface at the beginning of the simulations. **a**, Structural comparison of sHVF18 from simulations with lipid A aggregate (left, pink) and sHVF18 from NMR derived in TFE (right, green). A clustering analysis with an RMSD cut-off of 0.4 nm was performed on concatenated MD trajectories and representative structures of the top ten clusters (representing 92% of all simulations) are aligned and compared to the NMR structural ensemble. The staple is shown in grey. **b**, Average root mean square deviation (RMSD) of the backbone atoms of sHVF18 during the simulations after least square fit to the the NMR ensemble structures. Average values from three independent repeats are shown as a thick line, while the shaded area depicts the standard deviation. **c**, Average number of residues in the peptide forming an alpha helix during the simulations. The number of residues forming alpha helix in the NMR structure is shown by the dotted red line. **d**, A representative structure of the peptide-lipid complex. Lipids are shown in van der Waals representation with

lipid tails in grey and headgroups in dark green (phosphorus atoms) and red (oxygen atoms). Key residues involved in peptide binding are highlighted in stick representation and labeled. Ca^{2+} ions are shown as orange spheres. **e**, Average percentage of contacts made by each residue of the peptide with lipid tails, headgroup and Ca^{2+} ions during the simulations. Contacts with the phosphate group are highlighted in red. Error bars indicate standard deviations between repeat simulations. The cut-off distance for contact analysis is 0.4 nm.

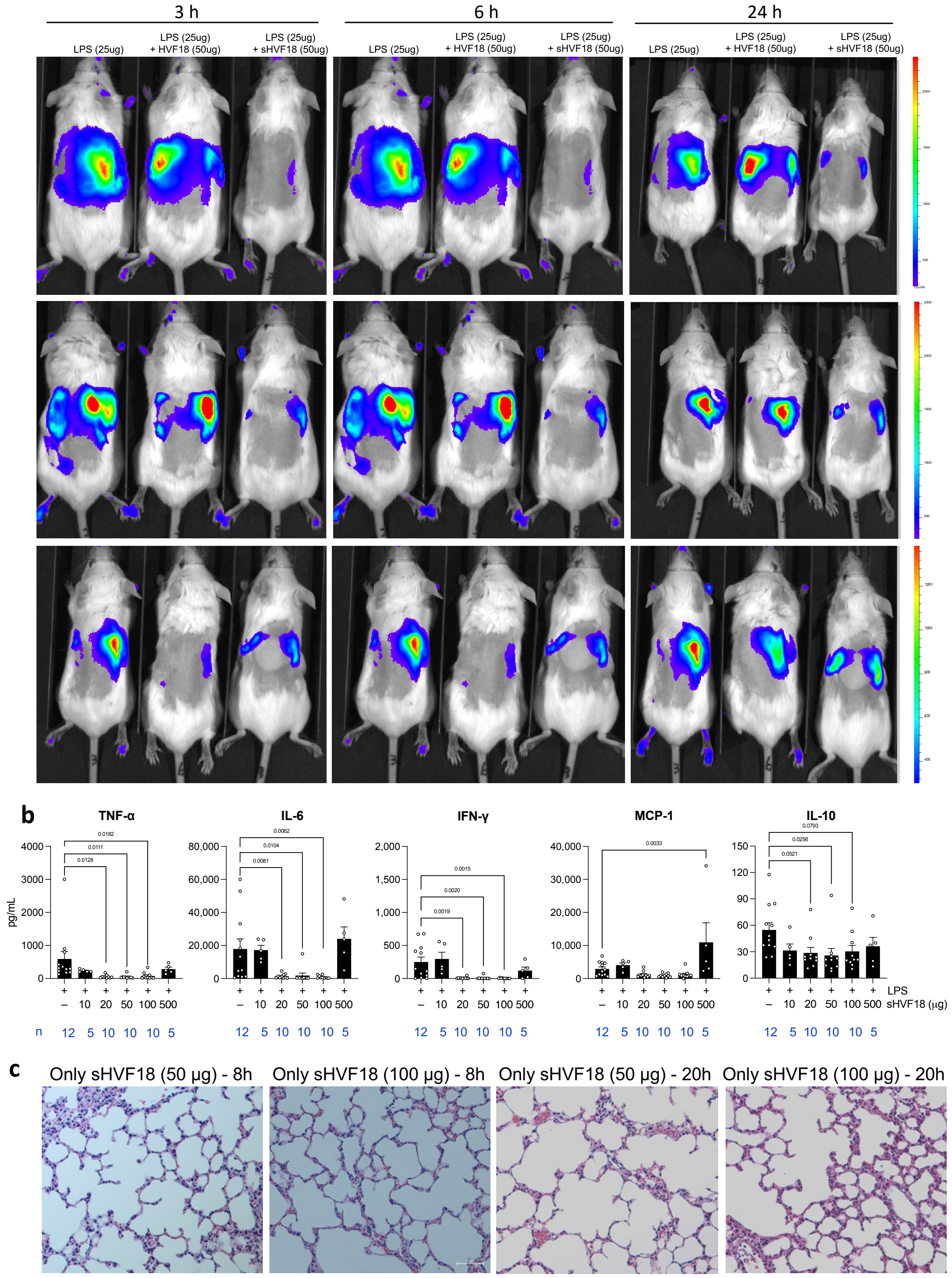

Supplementary Fig. 19 | Effects of the linear and stapled peptide on endotoxin responses in experimental mouse models. a, Representative *in vivo* inflammation imaging by IVIS in NF-κB reporter mice. HVF18 or sHVF18 (50 μg) were mixed with 25 μg LPS immediately before subcutaneous injection on the back of transgenic BALB/c Tg(NF-κB-RE-luc)-Xen reporter mice. *In vivo* imaging was acquired using an IVIS Spectrum bioimaging system at 3, 6, and 24 h after subcutaneous deposition. **b**, Cytokines release from plasma collected after 20

h from C57BL/6 mice stimulated with a sublethal dose of LPS and then treated with increasing doses of sHVF18. Data are presented as the means ± SEM, n represents the number of mice. P-values were determined using ordinary one-way ANOVA following Dunnett's multiple comparisons tests. c, Representative images show H&E staining of mouse lung tissue at 8 and 20 h in a mouse model treated with only sHVF18.

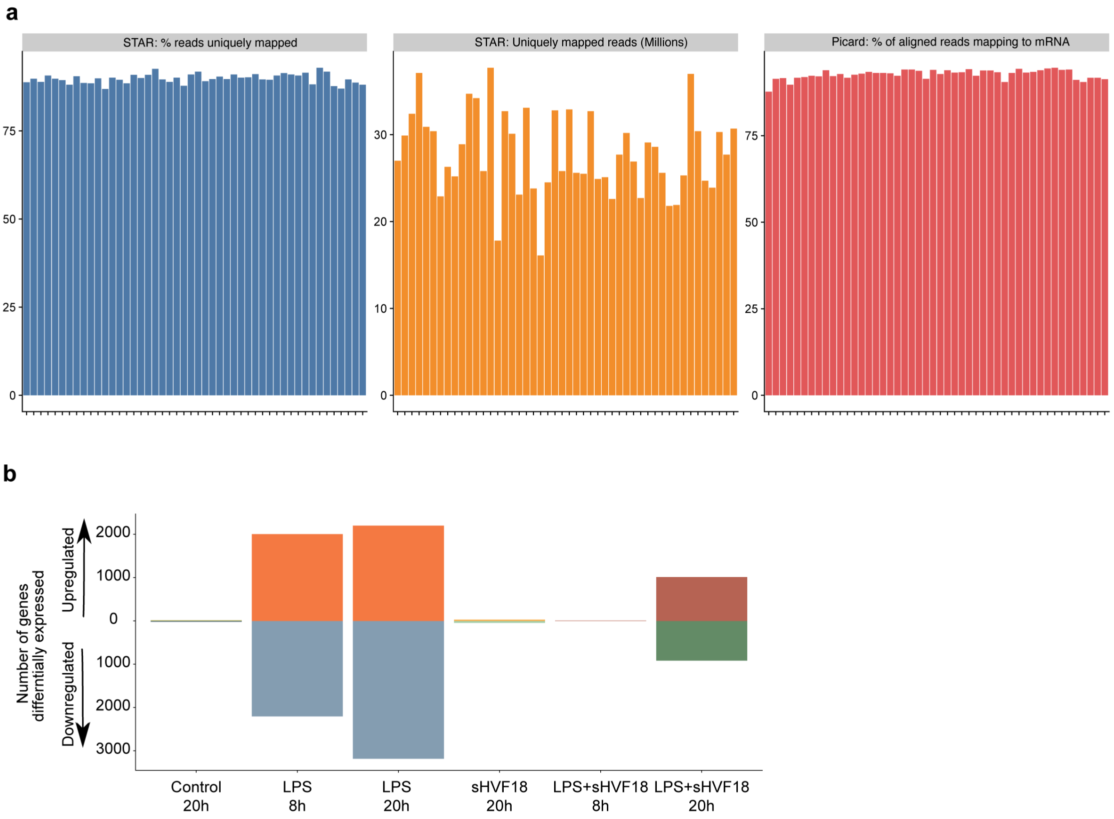

Supplementary Fig. 20 | Effect of sHVF18 on transcriptome profiling in mice stimulated with LPS. a, Quality metrics for RNA-seq samples. Bar plots show the percentage of reads mapping to the genome, millions of reads mapped per sample and the fraction of base pairs mapped to exonic/UTR regions of the genome. **b**, Number of differentially expressed genes compared to control (buffer only) 8 h.

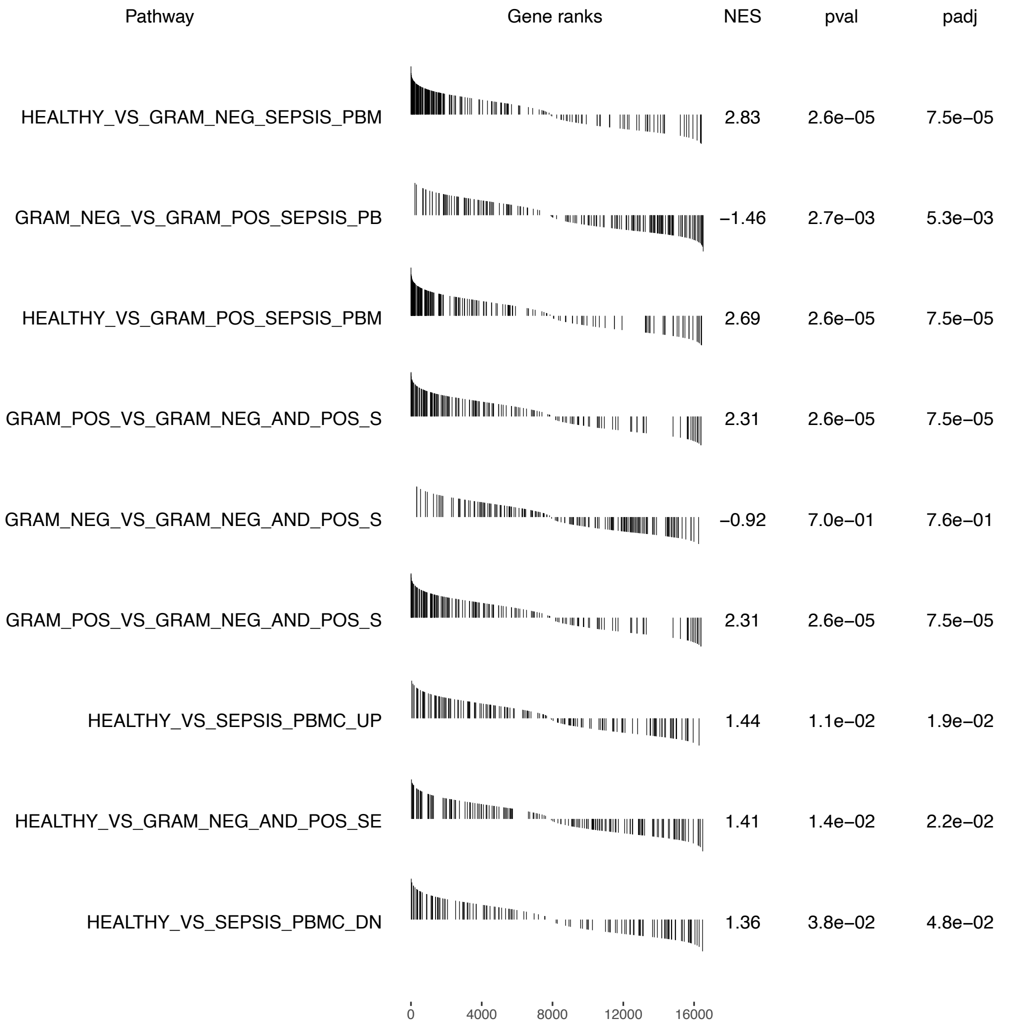

Supplementary Fig. 21 | Gene set enrichment analysis. Gene set enrichment analysis of 14 gene sets related to sepsis from the "immunologic signature gene sets" database (MSigDB, version 7.3) in LPS 8h compared to controls.

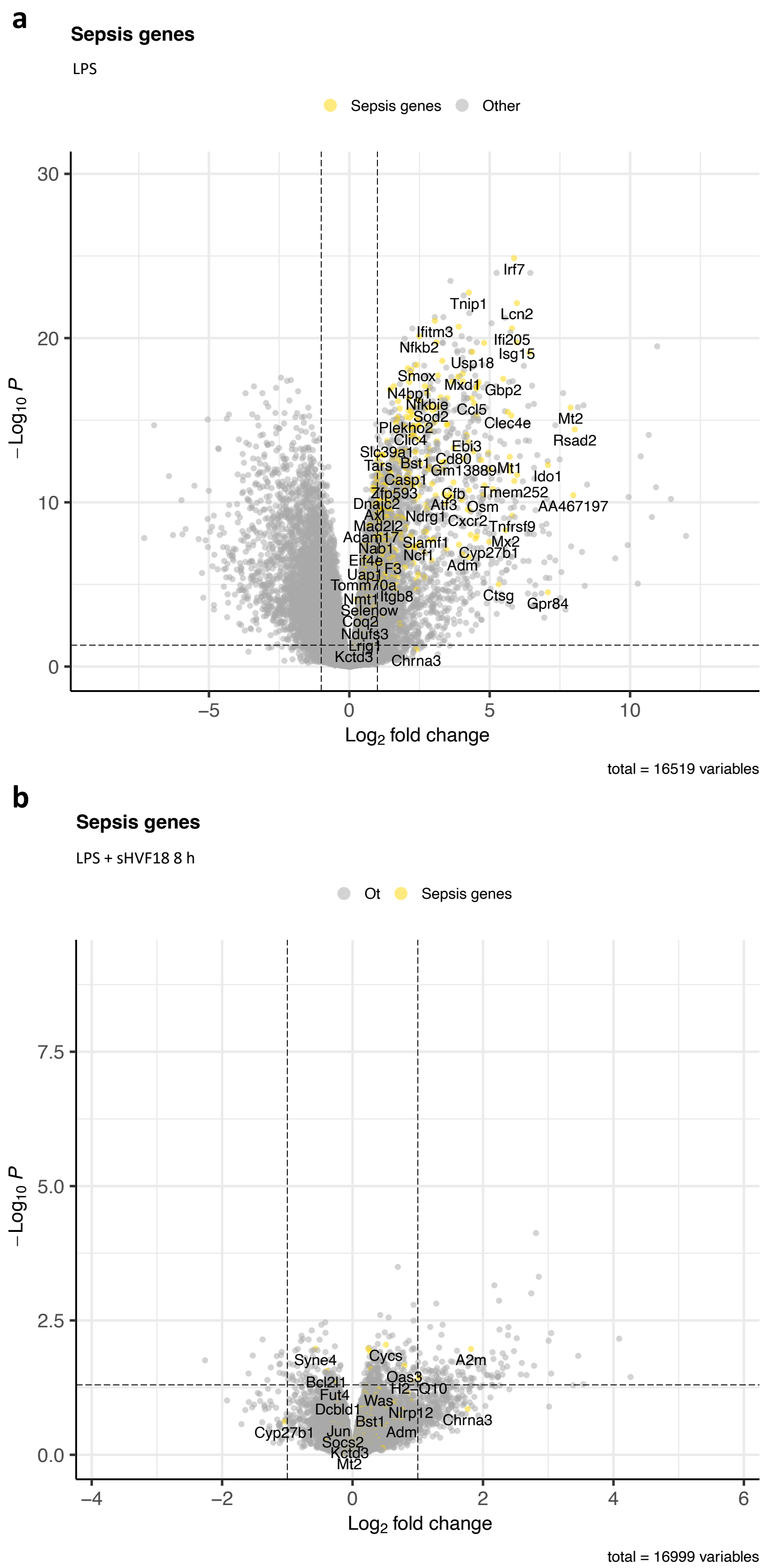

Supplementary Fig. 22 | Volcano plots. a, Volcano plot of LPS compared to control with leading edge genes for sepsis-related genes marked in yellow. **b**, Volcano plot of LPS + sHVF18 compared to control with leading edge genes for sepsis-related genes marked in yellow.

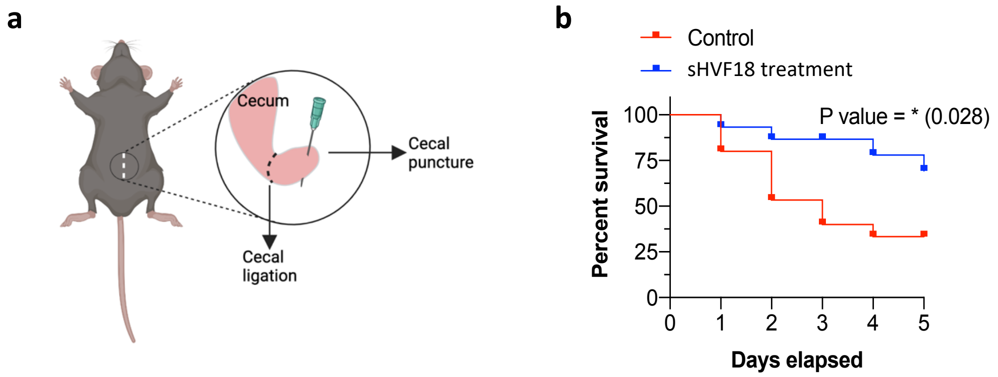

Supplementary Fig. 23 | Effect of sHVF18 treatment in a mouse CLP sepsis model. a, A standard CLP model of sepsis was employed using C57BL/6 mice. After the CLP procedure, the mice were i.p. treated twice daily with 100 µg of sHVF18 (in 100 µL water) for a total of 5 days. The first dosage was given 30 min after the CLP procedure. The control group was given 100 µL of water i.p. Figure created with BioRender.com. **b**, Kaplan-Meier survival curve of the percent survival within 5 days. sHVF18 treatment significantly improved the survival of mice. The p-value was determined using the Kaplan-Meier method (n = 10 mice/group).

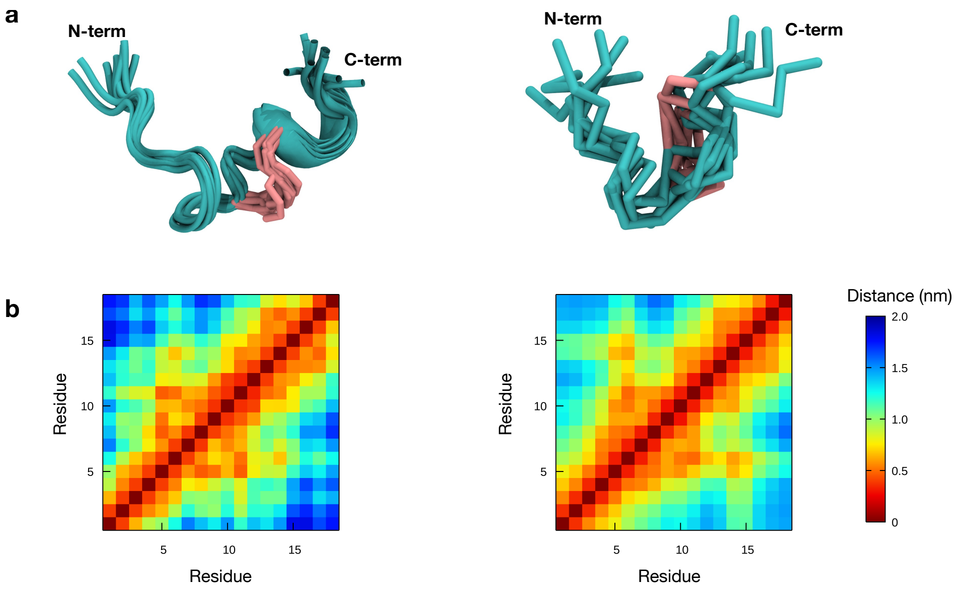

Supplementary Fig. 24 | Comparison of conformational sampling of the sHVF18 peptide in atomistic and CG simulations. a, Overlaid snapshots are taken every 500 ns from 5 µs atomistic (left) and CG (right) simulations of the peptide in solution. The atomistic peptide is shown in the cartoon representation, and the CG peptide is shown in the stick representation. For clarity, only the backbone of the peptide is shown. The staple is shown in pink, and the rest of the peptide is in cyan. **b**, Pair-wise distance between the Cα atoms in the atomistic peptide (left) and the backbone particles in CG peptide (right) averaged over the course of the simulations.

Supplementary References

1. Kasetty, G. et al. Structure-activity studies and therapeutic potential of host defense peptides of human thrombin. *Antimicrob Agents Chemother* **55**, 2880-2890 (2011).
2. Saravanan, R. et al. Structural basis for endotoxin neutralisation and anti-inflammatory activity of thrombin-derived C-terminal peptides. *Nat Commun* **9**, 2762 (2018).
3. Holdbrook, D.A. et al. Influence of pH on the activity of thrombin-derived antimicrobial peptides. *Biochim Biophys Acta Biomembr* **1860**, 2374-2384 (2018).
4. Houmard, J. & Drapeau, G.R. Staphylococcal protease: a proteolytic enzyme specific for glutamoyl bonds. *Proc Natl Acad Sci U S A* **69**, 3506-3509 (1972).
5. van der Plas, M.J. et al. Pseudomonas aeruginosa elastase cleaves a C-terminal peptide from human thrombin that inhibits host inflammatory responses. *Nat Commun* **7**, 11567 (2016).
6. Roccatano, D., Colombo, G., Fioroni, M. & Mark, A.E. Mechanism by which 2,2,2-trifluoroethanol/water mixtures stabilize secondary-structure formation in peptides: a molecular dynamics study. *Proc Natl Acad Sci U S A* **99**, 12179-12184 (2002).
