# Supplementary material for: Targeting Toll-like receptor-driven systemic inflammation by engineering an innate structural fold into drugs
Source: Nat Commun. 2023 Sep 29;14:6097. doi: 10.1038/s41467-023-41702-y (PMC10541425; doi:10.1038/s41467-023-41702-y)
Supplement: Supplementary file 3 — Description of Additional Supplementary Files [file 41467_2023_41702_MOESM3_ESM.pdf]

Description of Additional Supplementary Files

Supplementary Data 1

Raw HDX Experimental details and data analysis.

Supplementary Data 2

NMR Structure and NMR Structure Validation.
