# Supplementary material for: Targeting Toll-like receptor-driven systemic inflammation by engineering an innate structural fold into drugs
Source: Nat Commun. 2023 Sep 29;14:6097. doi: 10.1038/s41467-023-41702-y (PMC10541425; doi:10.1038/s41467-023-41702-y)
Supplement: Supplementary file 6 — Reporting Summary [file 41467_2023_41702_MOESM6_ESM.pdf]

nature portfolio

Corresponding author(s): Petruk Ganna

Last updated by author(s): Aug 31, 2023

Reporting Summary

Nature Portfolio wishes to improve the reproducibility of the work that we publish. This form provides structure for consistency and transparency in reporting. For further information on Nature Portfolio policies, see our Editorial Policies and the Editorial Policy Checklist.

Statistics

For all statistical analyses, confirm that the following items are present in the figure legend, table legend, main text, or Methods section.

n/a	Confirmed	
☐	☒	The exact sample size (*n*) for each experimental group/condition, given as a discrete number and unit of measurement
☐	☒	A statement on whether measurements were taken from distinct samples or whether the same sample was measured repeatedly
☐	☒	The statistical test(s) used AND whether they are one- or two-sided<br>*Only common tests should be described solely by name; describe more complex techniques in the Methods section.*
☒	☐	A description of all covariates tested
☐	☒	A description of any assumptions or corrections, such as tests of normality and adjustment for multiple comparisons
☐	☒	A full description of the statistical parameters including central tendency (e.g. means) or other basic estimates (e.g. regression coefficient) AND variation (e.g. standard deviation) or associated estimates of uncertainty (e.g. confidence intervals)
☐	☒	For null hypothesis testing, the test statistic (e.g. *F*, *t*, *r*) with confidence intervals, effect sizes, degrees of freedom and *P* value noted<br>*Give P values as exact values whenever suitable.*
☒	☐	For Bayesian analysis, information on the choice of priors and Markov chain Monte Carlo settings
☒	☐	For hierarchical and complex designs, identification of the appropriate level for tests and full reporting of outcomes
☒	☐	Estimates of effect sizes (e.g. Cohen's *d*, Pearson's *r*), indicating how they were calculated

Our web collection on statistics for biologists contains articles on many of the points above.

Software and code

Policy information about availability of computer code

Data collection

Circular dichroism, Spectra Manager™ Suite Spectroscopy Software - Jasco Inc.
Gel electrophoresis, ChemiDoc™ Imaging System (Bio-Rad).
HPLC, Open LAB CDS ChemStation from Agilent Technologies AB.
LC-MS/MS, HFX Orbitrap MS system (Thermo Scientific) equipped with a Dionnex 3000 Ultimate HPLC (Thermo Fisher).
Absorbance at different wavelength, Wallac 1420 Workstation Sofrware version 3.00.
HDX-MS analysis, LEAP H/D-X PAL™ platform interfaced to an LC-MS system, comprising an Ultimate 3000 micro-LC coupled to an Orbitrap Q Exactive Plus MS.
NMR analysis, 700 MHz Bruker Avance III HD spectrometer equipped with QCI cryo-probe and pulse field gradients.
Microscale thermophoresis (MST) was performed on a NanoTemper Monolith NT.115 apparatus (Nano Temper Technologies).
In Vivo Imaging System, IVIS Spectrum, (PerkinElmer Life Sciences).
Histology, Olympus CKX53 microscope.
SEM images, Jeol JSM-7800F FEG-SEM.
RNA-seq: NovaSeq 6000 System (Illumina).
Pig polmunary parameters, Ventilator Siemens-Elema (Servo 900C), PexA 2.0 instrument was used for hemodynamic parameters.

Data analysis

Statistical data, GraphPad Prism v8.0.
MS/MS spectra were searched with PEAKS (version 10) against UniProt Homo Sapiens (version 2020_02).
HDX analysis, PEAKS Studio X Bioinformatics Solutions Inc., HDExaminer, version 3.3.0.

NMR structure calculation, GROMACS molecular dynamics suite using the AMBER99SB force field, CCPNMR suite , and MolProbity software suite.
Kd from Microscale Thermoforesis, MO.Affinity Analysis Software.
Integrative modeling of human CD14, Modeller version 9.21, CHARMM-GUI Solution Builder.
In silico analysis, ClusPro web server.
Molecular dynamics simulation, CHARMM-GUI Solution Builder, GROMACS 2021 and the CHARMM force field.
Bioluminescence from the mice, Living Image 4.0 Software (PerkinElmer Life Sciences).
Transcriptomic data, aligned to the GRCm38 genome using gencode version 25 as the gene model using STAR (2.7.6a). Quality control was performed by collating data from STAR and Picard with no sample failing quality control. Gene quantification was done using featureCounts. 979 For downstream analysis, R (version 4.0.3) was used, and plotting was done using ggplot2 (2.3). Pre-processing was done using the voom-function (limma version 3.46). Transcription factor and GO enrichment analysis was done using the clusterProfiler package (3.18) using MSigDB collections C3 and C5. A Venn diagram was plotted using eulerr.

For manuscripts utilizing custom algorithms or software that are central to the research but not yet described in published literature, software must be made available to editors and reviewers. We strongly encourage code deposition in a community repository (e.g. GitHub). See the Nature Portfolio guidelines for submitting code & software for further information.

Data

Policy information about availability of data

All manuscripts must include a data availability statement. This statement should provide the following information, where applicable:

- Accession codes, unique identifiers, or web links for publicly available datasets
- A description of any restrictions on data availability
- For clinical datasets or third party data, please ensure that the statement adheres to our policy

The original experimental data that support the findings of this work are available as Source Data 1 for the main figures, and as Source Data 2 for the Supplementary Information.
The MS raw files have been deposited to the ProteomeXchange Consortium via the MassIVE partner repository (reference ID: MSV000090815, [https://massive.ucsd.edu/ProteoSAFe/dataset.jsp?task=20d9f7e7f6434abfa3d56e0bf4eedf99]. The MassIVE DOI for this deposition is 10.25345/C5KD1QQ7S.
The molecular dynamics trajectories for the initial and final configurations have been deposited in the Zenodo database [https://zenodo.org/record/8267913]. The Zenodo DOI for this deposition is 10.5281/zenodo.8267913.
The NMR data have been deposited to the wwPDB. The PDB code for this deposition is 8BWW. The PDB DOI for this deposition is 10.2210/pdb8bww/pdb. The BMRB code for this deposition is 34778.
PDB data: 4GLP [https://www.rcsb.org/structure/4GLP], 1WWL [https://www.rcsb.org/structure/1WWL], 5Z5X [https://www.rcsb.org/structure/5Z5X].

Human research participants

Policy information about studies involving human research participants and Sex and Gender in Research.

Reporting on sex and gender	The study design did not specifically consider sex and gender as a factor. However, since sex is known to influence immune response, in experiments with human blood, a balanced representation of both sexes was insured. No separate data analysis for males and females was performed.
Population characteristics	Age range was 22 to 50 years, but it was not considered a covariate in this study as it is not expected to affect the results. Other demographic variables were not collected as our ethical permission did not allow this.
Recruitment	Participants were recruited on a volunteer basis from the research center. We do not expect this selection process to bias the results.
Ethics oversight	The use of blood was approved by the Ethics Committee at Lund University, Lund, Sweden (permit number: DNR2015/801).

Note that full information on the approval of the study protocol must also be provided in the manuscript.

Field-specific reporting

Please select the one below that is the best fit for your research. If you are not sure, read the appropriate sections before making your selection.

☒ Life sciences ☐ Behavioural & social sciences ☐ Ecological, evolutionary & environmental sciences

For a reference copy of the document with all sections, see nature.com/documents/nr-reporting-summary-flat.pdf

Life sciences study design

All studies must disclose on these points even when the disclosure is negative.

Sample size	No statistical methods were used to predetermine sample size estimates. Sample size was determined based on standards in the field and experiments to obtain statistical significance and reproducibility. At least triplicates were used to meet the minimal requirements for

statistical analysis and the detailed sample size was demonstrated in the figure legends.
Relevant references:

1. Puthia, M., Butrym, M., Petrlova, J., Stromdahl, A.C., Andersson, M.A. et al. A dual-action peptide-containing hydrogel targets wound infection and inflammation. Sci Transl Med 12 (2020).
2. van der Plas, M.J., Bhongir, R.K., Kjellstrom, S., Siller, H., Kasetty, G. et al. Pseudomonas aeruginosa elastase cleaves a C-terminal peptide from human thrombin that inhibits host inflammatory responses. Nat Commun 7, 11567 (2016).
3. Papareddy, P., Rydengard, V., Pasupuleti, M., Walse, B., Morgelin, M. et al. Proteolysis of human thrombin generates novel host defense peptides. PLoS Pathog 6, e1000857 (2010).
4. Mourtada, R., Herce, H.D., Yin, D.J., Moroco, J.A., Wales, T.E. et al. Design of stapled antimicrobial peptides that are stable, nontoxic and kill antibiotic-resistant bacteria in mice. Nat Biotechnol 37, 1186-1197 (2019).
5. Buras, J.A., Holzmann, B. & Sitkovsky, M. Animal models of sepsis: setting the stage. Nat Rev Drug Discov 4, 854-865 (2005).
6. Rittirsch, D., Huber-Lang, M.S., Flierl, M.A. & Ward, P.A. Immunodesign of experimental sepsis by cecal ligation and puncture. Nat Protoc 4, 31-36 (2009).
7. Ghaidan, H., Stenlo, M., Niroomand, A., Mittendorfer, M., Hirdman, G. et al. Reduction of primary graft dysfunction using cytokine adsorption during organ preservation and after lung transplantation. Nat Commun 13, 4173 (2022).
8. Stenlo, M., Hyllen, S., Silva, I.A.N., Bolukbas, D.A., Pierre, L. et al. Increased particle flow rate from airways precedes clinical signs of ARDS in a porcine model of LPS-induced acute lung injury. Am J Physiol Lung Cell Mol Physiol 318, L510-L517 (2020).

Data exclusions: No data was excluded from analysis.

Replication: To ensure the replication of the findings, experiments were repeated at different times as indicated in the figure legends. All experimental data was reliably reproduced in multiple independent experiments. For in vivo experiments, multiple mice and pigs were used to ensure reproducibility, the exact number of animals was shown in the figure legends or as dots in the graphs.

Randomization: No randomization was used in this study. Covariates such as batch variation in animals was controlled by testing the different conditions in each batch. To avoid bias in selection of more or less healthy animals to the treatment groups, the treatment was assigned before the inflammation was induced.

Blinding: The experiments were not blinded because the appearance of the two solutions was different and so blinding was not feasible. Data analysis for in vivo studies was performed blindly.

Reporting for specific materials, systems and methods

We require information from authors about some types of materials, experimental systems and methods used in many studies. Here, indicate whether each material, system or method listed is relevant to your study. If you are not sure if a list item applies to your research, read the appropriate section before selecting a response.

Materials & experimental systems

n/a	Involved in the study
☐	☒ Antibodies
☐	☒ Eukaryotic cell lines
☒	☐ Palaeontology and archaeology
☐	☒ Animals and other organisms
☒	☐ Clinical data
☒	☐ Dual use research of concern

Methods

n/a	Involved in the study
☒	☐ ChIP-seq
☒	☐ Flow cytometry
☒	☐ MRI-based neuroimaging

Antibodies

Antibodies used: The following anti-human antibodies were used :
Human inflammation DuoSet® ELISA Kit (R&D Systems, Minneapolis, MN, USA) specific for TNF-a (Cat. N. DY210, Lot. P258109 and Lot. P198969).
Human inflammation DuoSet® ELISA Kit (R&D Systems, Minneapolis, MN, USA) specific for IL-1beta (Cat. N. DY201, Lot. P275844 and Lot. P225120).

The following anti-mouse antibodies were used :
Mouse Inflammation Kit (Becton Dickinson AB, Cat. N. 552364) containing antibodies for TNF- a, IFN-gamma, MCP-1, IL-10, and IL-6.

Validation: All antibodies were used according to the manufacturer's instructions.

Eukaryotic cell lines

Policy information about cell lines and Sex and Gender in Research

Cell line source(s): THP1-XBlue-CD14 reporter cells were purchased from InvivoGen (San Diego, CA, USA).

Authentication	THP1-XBlue-CD14 reporter cells were authenticated by the manufacturer (InvivoGen, San Diego, CA, USA). No further validation was performed.
Mycoplasma contamination	We have confirmed that the cells were tested negative for mycoplasma contamination.
Commonly misidentified lines (See ICLAC register)	We did not use a commonly misidentified cell line in this study.

Animals and other research organisms

Policy information about studies involving animals; ARRIVE guidelines recommended for reporting animal research, and Sex and Gender in Research

Laboratory animals	BALB/c tg(NF-kB-RE-Luc)-Xen reporter mice (Taconic, 10–12 weeks old).<br>C57BL/6 (Janvier, 7-8 and 11–12 weeks old).<br>Farm-raised wild-type American Yorkshire pigs (Sus scrofa domesticus).
Wild animals	No wild animal were involved in this study.
Reporting on sex	Gender was not considered in the data analysis.
Field-collected samples	Not involved.
Ethics oversight	All experiments on mice were performed according to Swedish Animal Welfare Act SFS 1988:534 and were approved by the Animal Ethics Committee of Malmö/Lund, Sweden (permit numbers M8871-19, 16542-21).<br>The study on pigs was approved by the local Ethics Committee for Animal Research (DNR 5.2.18-4903/16, and DNR 5.2.18-8927/16) at Lund University. All animals received care according to the USA Principles of Laboratory Animal Care of the National Society for Medical Research, Guide for the Care and Use of Laboratory Animals, National Academies Press (1996).

Note that full information on the approval of the study protocol must also be provided in the manuscript.
